# Neuronal signature of spatial decision-making during navigation by freely moving rats by using calcium imaging

**DOI:** 10.1073/pnas.2212152119

**Published:** 2022-10-24

**Authors:** Francesco Gobbo, Rufus Mitchell-Heggs, Dorothy Tse, Meera Al Omrani, Patrick A. Spooner, Simon R. Schultz, Richard G. M. Morris

**Affiliations:** ^a^Centre for Discovery Brain Sciences, Edinburgh Neuroscience, The University of Edinburgh, Edinburgh, EH8 9JZ, UK;; ^b^Department of Bioengineering and Centre for Neurotechnology, Imperial College London, London, SW7 2AZ, UK;; ^c^Department of Psychology, Edge Hill University, Ormskirk, L39 4QP, UK;; ^d^MSc Program in Integrative Neuroscience, University of Edinburgh, Edinburgh, EH8 9JZ, UK;; ^e^Simons Initiative for the Developing Brain, University of Edinburgh, Edinburgh, EH8 9JZ, UK

**Keywords:** hippocampus, place cells, spatial navigation

## Abstract

A challenge in spatial memory is understanding how place cell firing contributes to decision-making in navigation. A spatial recency task was created in which freely moving rats first became familiar with a spatial context over several days and thereafter were required to encode and then selectively recall one of three specific locations within it that was chosen to be rewarded that day. Calcium imaging was used to record from more than 1,000 cells in area CA1 of the hippocampus of five rats during the exploration, sample, and choice phases of the daily task. The key finding was that neural activity in the startbox rose steadily in the short period prior to entry to the arena and that this selective population cell firing was predictive of the daily changing goal on correct trials but not on trials in which the animals made errors. Single-cell and population activity measures converged on the idea that prospective coding of neural activity can be involved in navigational decision-making.

## Significance

This manuscript addresses a longstanding problem about how place cell firing contributes to navigation in spatial environments, and data derived from on-line calcium imaging offer a new solution. These data support the view that prior to entering an arena where an animal will navigate to only one of several possible places on each day, a population of spatially responsive cells, including place cells, fire during the last 5 s. This population is shown to be predictive of the destination and trajectory that the animal is about to take.

A challenge in understanding spatial memory and spatial navigation is explaining how the former contributes to the latter. It is generally accepted that place cells (PCs) in the hippocampus constitute a “cognitive map” of the external space and that maps are used for navigation (1, 2). An outstanding question is how these two aspects can be linked—how information stored in the map can be accessed to guide choices about navigation. Numerous neurobiological studies have shown that laboratory animals readily learn to direct their spatial navigation accurately from one known place to another. This has been observed in diverse tasks such as the Barnes Maze (3) and the watermaze (4), with accurate navigation lost after hippocampal lesions. The cognitive map theory defines hippocampal PCs as active only in a particular position in a specific environment. How then can PCs corresponding to distal places A, B, or C contribute to appropriate navigation when the animal is starting his navigation from a remote start place S?

One solution might be to represent the animal’s location in vectorial terms with respect to relevant landmarks or goals. This has found support in the discovery of object vector cells in the rat entorhinal cortex (5) and goal-oriented vector fields of PCs in the hippocampus of bats (6) and, recently, of rats (7). Modulation of in-place activity has been reported depending on future paths or goal locations (8–10). However, this solution may not explain the decision-making aspect entailed in flexible choices, such as the decision to go from place S to place A but not to other places. With respect to decision-making, one suggestion is that occasional out-of-field neural activity might be used. For example, the look-ahead activity of hippocampal CA3 PCs at decision choice points has been reported in rats running in figure-of-8 mazes (11, 12). Activity replay, a phenomenon first identified during sleep (13) or rest (14) after daytime experience, was later found to be involved in the out of-field reactivation of PCs during awake navigation to a learned home-goal from random locations (15). Replay could therefore be a possible way for the brain to selectively accesses spatial information beyond the current location. It could sometimes reflect intended navigation and has therefore been labeled as prospective replay. The role of replay during decision-making has been actively debated in the literature, with different groups suggesting that replayed events may reflect future paths (15, 16), paths to be avoided (17), new or updated learning (18), or the remembering of past trajectories (19). Hence, we sought to observe the neuronal signature of decision-making when rats learn and use newly acquired information to navigate to distinct locations across different sessions.

Four key issues guided the experimental design. The first, following Pfeiffer and Foster (15), was to visually monitor enough cells simultaneously in an individual animal because only a subset may participate in navigational planning. The second was to be sure of recording from the same cells across days in order that firing on one day, when the animal may learn to go to A, can be compared to that seen on another day when it encodes the new location that day and remembers to go to B or C but not A. The widely used multiple single-cell tetrode recording technique addresses the first issue with excellent temporal resolution but is less suitable for the second because one cannot be certain that a specific cell on day N is also recorded on day N+1. Indeed, it is not uncommon for tetrodes to be advanced slightly from day to day in search of additional cells. The third issue was to devise a suitable behavioral task for the navigation problem in which the animal must encode which of the three distinct targets in a familiar spatial context is correct on any day and direct its navigation appropriately to the most recent correct location. The fourth issue is to ensure that the chosen technique does not pose too heavy a constraint on the behavioral protocol, with behavioral performance being minimally influenced (20). The present study meets all four challenges. It uses an event arena in which rats are trained over many days (28 or more) and thereby become familiar with the testing context. They develop a stable spatial map whose PCs can be repeatedly mapped during a daily 10-min exploration phase. The target location of A, B, or C of the animal’s navigation from a start location S was varied from day to day. In effect, the daily task is a spatial “recency” task in which one location, but not the other two, is identified as the rewarded target for that session. The study deploys calcium (Ca^2+^) imaging, which has recently been used to monitor neuronal activity during learning and neuronal reactivation (21, 22), as the recording technique of choice. This provides high cell numbers and ensures day-to-day reproducibility and stability, albeit at the expense of temporal resolution. The Ca^2+^-transients were monitored using a gradient refractive-index (GRIN) lens directed at area CA1 of the hippocampus in freely moving rats. Using both single-cell and manifold population analyses of cell firing, we reveal a solution to the navigation problem.

## Results

### Calcium Imaging in Rat CA1.

**Fig. 1.**
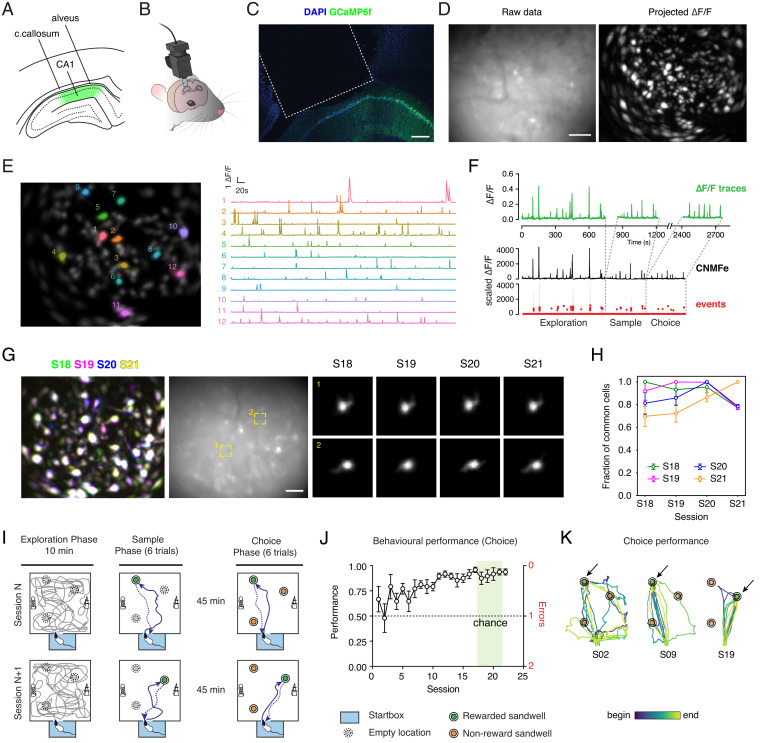
Miniscope calcium imaging in rat CA1. (*A*) Schematic of rat hippocampus CA1 area. The hippocampus is overlaid dorsally by alveolar fibers and callosal fibers. GCaMP6f is expressed in CA1 pyramidal neurons (depicted in green). (*B*) The GRIN lens is implanted to reach the outer surface of CA1 and cemented to the animal’s skull. A baseplate is attached externally to allow the daily positioning of the miniscope and recordings from the same neurons across multiple sessions. (*C*) Histological analysis confirmed the positioning of the GRIN lens as above stratum oriens of CA1. In green, own GCaMP6f fluorescence; in blue, DAPI staining of nuclei. Scale bar, 250 μm. The dashed line indicates the position occupied by the GRIN lens. (*D*) FOV of rat CA1 seen as recorded with the miniscope. The image displays the green fluorescence seen through the 535/50 filter from 475-nm illumination; note the occasional blood vessels and some brighter or active cells in the frame. Scale bar, 100 μm. On the *Right*, projected ΔF/F of the video on the *Left*. After processing, the signal is normalized in ΔF/F units, and maximum projection is shown to display active cells. (*E*) The CNMF-E algorithm was used to identify neurons. Representative temporal traces for 12 cells of the animal in *D*; note that the beginning of the recording is shown. The corresponding cells are highlighted on the cell map on the *Left*. (*F*) Event detection was performed with the OASIS algorithm from CNMF-E traces. The calculated ΔF/F profile of the applied mask is shown to compare the data traces with the CNMF-E output. (*G*) Longitudinal registration across consecutive sessions (S18 to S21). Superposition of the projected ΔF/F recordings for the four sessions confirms the consistency of the FOV. In the *Middle*, the corresponding FOV is presented; scale bar, 100 μm. In the two *Insets*, two representative cells are shown as they are identified in the four sessions. (*H*) Pairwise comparison of common cells between sessions. A large proportion of cells is detected and identified across multiple sessions. Average ± SEM, *n* = 6 animals. Note that the comparison of each session with itself is 1 by definition. (*I*) Schematic of the behavioral task in the everyday arena. Each session is made of three phases, as follows: exploration in the arena (10 min), sample phase (6 trials), and choice phase (6 trials). The text and *Materials and Methods* contain details. The startbox location from which the animal starts and then returns to eat the rewards is depicted in light blue. Each session, the identity of the rewarded sandwell changes. Ca^2+^ recordings are typically longer and are synchronized with the behavioral video with a transistor-transistor logic (TTL) pulse. (*J*) Behavioral performance increased over sessions, as animals committed fewer errors in identifying the correct sandwell during choice trials. Average ± SEM. For each animal, the average number of errors in the first three choice trials is expressed as performance that assumes a value of 0.5 at chance (*Materials and Methods*). The sessions highlighted in green are the recorded sessions considered in this work. Session effect, *P* < 0.05, one-way ANOVA. Here and in all later instances, full statistical details are reported in *SI Appendix*, Table S1. (*K*) Example trajectories of an animal performing in the choice phase at different stages of learning (S02, S09, and S19). Note how trials become more directed and fewer errors are committed.

To record from freely moving rats performing the spatial memory/navigational task, we used miniature endoscopes (miniscopes) originally introduced by the Schnitzer group (23, 24). Successful recordings in CA1 have so far been performed in mice, but we sought to adapt the technique to rats and were successful in recording from hundreds of cells per animal (in this study, 1,146 neurons from 6 animals, mean = 191 ± 43 SEM). A key step required in rat was to aspirate beyond the fibers of the corpus callosum to invade also some of the myelinated fibers of the alveus that would otherwise limit the visibility of CA1 neurons from the GRIN lens (Fig. 1*A* and *SI Appendix*, Fig. S1). This was achieved with care resulting in minimal damage to stratum oriens. When the miniscope was fitted to its baseplate on each day (Fig. 1*B*), its view through the GRIN lens that had been lowered during surgery to the outer surface of the hippocampus allowed the imaging of Ca^2+^-transients from pyramidal CA1 neurons (Fig. 1*C* and *SI Appendix*, Fig. S2). It proved possible to record stably from large numbers of neurons simultaneously with high signal to noise (*SI Appendix*, Fig. S2*A*). After the raw camera videos were processed (Fig. 1 *D*, *Left*), recordings were processed to identify individual regions of interest (ROIs; *Materials and Methods*) with the CaImAn constrained nonnegative matrix factorization-extended (CNMF-E) algorithm (Fig. 1 *D*, *Right*). The Ca^2+^ activity associated with individual ROIs, which likely corresponded to single cells, could be readily distinguished (Fig. 1*E*) and monitored during distinct exploration, sample, and choice phases of the daily behavioral task (see below). On the top rows of Fig. 1*F*, the correspondence between CNMF-E traces and the fluorescence profile of the resulting ROIs expressed as ΔF/F can be seen. Individual Ca^2+^ events were detected from CNMF-E traces (Fig. 1*F*). An ostensible virtue of Ca^2+^ imaging is the ability to monitor neurons across days by matching corresponding ROIs and thus explore the consistency of cell firing and its behavioral and spatial correlates across training sessions (24, 25). We were able to obtain stable fields of view and record from the same cells across periods of 4 sessions (Fig. 1*G* and *SI Appendix*, Fig. S3*A*) and for as long as 30+ days (*SI Appendix*, Fig. S3*B*). This stability is shown for two illustrative cells (nominally called 1 and 2 in Fig. 1 *G*, *Middle*) across sessions 18 to 21 of training. Around 80% of neurons could be reliably found across sessions (Fig. 1*H*). We confirmed that our recordings came from excitatory neurons expressing the calcium sensor GCaMP6f (26) from the CamKII promoter. GCaMP6f expression overlapped with the pan-neuronal marker NeuN but not with the inhibitory neuron marker GAD67 (*SI Appendix*, Fig. S4). To our knowledge, only one recent study (27) has been published using single-photon calcium imaging in rat CA1. This confirmed the presence of PCs, and our findings build on theirs as an application of this technology to freely moving rats undertaking flexible spatial choice behavior.

### “Everyday Memory” Task.

The concept of everyday memory (28) is that a great deal of information is encoded incidentally during daily life that is typically retained selectively for not much more than a day. In our model of this phenomenon, food-restricted rats were trained during a sample phase to retrieve food pellets from a single sandwell in a square arena whose location changed daily. There was no requirement to learn, as it was not a trial requiring discrimination between two choices, but the animals were rewarded at the daily location that was encoded. This daily training was preceded each day by an exploration phase of the entire arena in which the 3 possible locations for this rewarded sandwell were initially empty (Fig. 1*I*). Access to the arena from a startbox (blue) was via an automated entry door. The exploration phase lasted 10 min during recording sessions. The sample phase (6 trials) then began in which the animals now found that one of the possible sandwell locations had sand in it (green in Fig. 1*I*) that, were the animal to dig, would reveal accessible food pellets. The food pellets are large (0.5 g) such that the animals have a natural disposition to carry them back to the startbox to eat. All animals quickly returned to the startbox after retrieving each pellet one by one. The other two possible sandwell locations were empty, with food pellets in an underlying inaccessible compartment to ensure uniform olfactory cues (29). After 45 min, the choice phase (6 trials) began in which the animals were now confronted by 3 identical-looking sandwells, of which only the one used in the sample phase contained an accessible food reward (win-stay). The animal’s path from the startbox around the arena and among the sandwells was monitored on both sample and choice trials. On the next session, one day later, the position of the correct sandwell changed to a new location in a quasi-random order. Over time, the probability that the animals would preferentially approach this sandwell during choice trials improved (F = 2.88, df = 21, *P* = 0.034; Fig. l) such that, by sessions 18 to 21 (shaded green; Fig. 1), the animals reached an asymptote of performance of 90.6 ± 3.5% correct (mean ± SEM, Student’s *t* test t = 11.61 relative to chance, df = 7, *P* < 0.0001; here and throughout the text, full statistics information is reported in *SI Appendix*, Table S1; Fig. 1*J*, and *SI Appendix*, Fig. S5). The time taken by the animal to reach the sandwell that was most recently correct also declined across sessions (*SI Appendix*, Fig. S5*A*). In the last 2 sessions, a probe trial was performed in which the sandwells were covered with sand but had no accessible food reward. The animals tended to focus their digging for food preferentially on the correct sandwell (correct-incorrect effect, *P* < 0.001, two-way ANOVA; *SI Appendix*, Fig. S5*C*). It is important to note that the learning curve of Fig. 1*J* is not gradual learning of the spatial map of the arena and the 3 possible sandwell locations; such context learning is likely accomplished in the earliest sessions. Instead, it is the acquisition of the principle of using memory recall to approach the most recently rewarded location (spatial recency memory). Fig. 1*K* plots the paths taken by a representative animal at different stages of learning (in this example, sessions 2, 9, and 19). As the animal became more proficient in the task, more direct paths were taken and fewer errors were committed. Thus, the behavioral task created an ideal daily neuronal recording session in which there is a defined starting location, three geometrically distinct targets, and the opportunity to follow directed navigational paths from the start to the most recently valued target and back home again. We therefore focused our attention on sessions 18 to 21 during which the animals’ asymptotic performance in the task was recorded using the miniscope.

### Spatial Properties of Cells Recorded with Ca^2+^ Imaging.

**Fig. 2.**
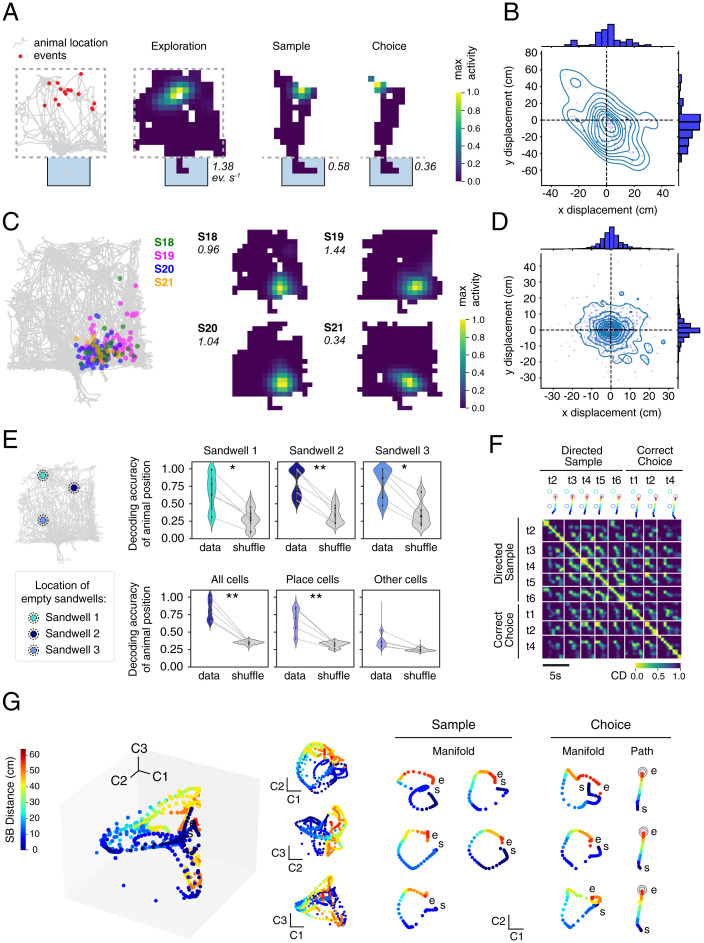
Features of space representation in rat CA1. (*A*) Example cell showing the consistency of event locations between exploration, sample, and choice. On the *Left*, the position of the events of the displayed cell (red dots) are superimposed on the animal’s trajectory. On its *Right*, the corresponding occupancy maps are shown for the three phases. The light-blue box is the startbox, and the dashed line indicates the arena limits. In italic is the peak value for each map (events/second). (*B*) x-y displacement of the maximum activity for all neurons in sample and choice phases compared to the exploration phase. (*C*) Individual cells display consistent location of their place fields across consecutive sessions. Displayed is one representative cell detected in S18 to S21. On the *Left* is shown the position of the events color coded by session. On the *Right*, corresponding occupancy maps are shown for the four sessions. (*D*) x-y displacement of the place center across exploration sessions. The first session where a PC is detected is taken as reference to calculate the displacements for the other sessions. (*E*) Decoding accuracy of a decoder trained on the exploration phase activity. On the *Left*, location and naming of the three sandwell locations used in this study. *Top*, the decoder correctly predicted when the animals were located in one of the three sandwells. Violin plot, points are individual animals. *P < 0.05, **P < 0.01, Student’s *t* test of data vs. shuffle comparison. *Bottom*, performance of the decoder using all of the cells detected (*Left*), only PCs (*Middle*), or other spatially sensitive cells (non-PCs, *Right*). **P < 0.01, Student’s *t* test of data vs. shuffle. *SI Appendix*, Table S1 shows for full statistics. (*F*) Cosine dissimilarity matrix for directed sample trials (trials 2, 3, 4, 5, and 6), and correct choice trials (trials 1, 2, and 3) showing repeated neuronal activity along the trajectory. Trajectories are represented on top of the graph. (*G*) Manifold representation of trials presented in *F*. The majority of variance is contained in the first two components C1 and C2. The s denotes the start of the trials in the startbox, and the e denotes the end of the outbound part of each trial at the sandwell. Note the repeated pattern assumed by the individual trial manifolds (*Right*) indicating the repetitiveness of the neural activity. The manifold assumes a circular shape even though it only corresponds to the outbound trajectory between the startbox and the sandwell (shown for choice trials), indicating a link between the startbox activity and the sandwell representation.

The next step was to conduct Ca^2+^ imaging during all three phases of the task (exploration, sample, and choice; Fig. 2). We confirmed observations from mouse experiments that miniscope Ca^2+^ imaging enables the visualization of place fields in the event arena. They were observed in individual cells during all phases of the session (Fig. 2*A*). PCs have been identified in linear and multidimensional environments (30); we also consistently observed many neurons displaying spatial selectivity (*SI Appendix*, Fig. S6). Multiple factors including directed navigation may induce global or rate remapping (31). To determine if remapping was occurring between exploration and sample or choice phases, the center of place fields identified in the exploration phase were compared with the maximum activity during sample and choice phases; there was no trend toward either global or rate remapping, taking into account the lesser space navigated during the training trials (Fig. 2*B*). Moreover, by pooling data across sessions 18 to 21, we were able to observe a reasonably thorough mapping of the two-dimensional (2D) space of the entire event (*SI Appendix*, Fig. S6*C*).

Miniscope Ca^2+^ imaging offers the opportunity to examine stability between sessions (Fig. 2*C*). The quantification across sessions 18 to 21 showed most place-field centers displayed a spatial drift of 10 cm or less in the two arena axes (Fig. 2*D* and *SI Appendix*, Fig. S7), with the activity monitored across four separate exploration sessions of 10 min each. Thus, neurons did not significantly change the position of their place field over these four consecutive days. A neuron was considered a PC if the following four criteria were met: 1) the neuron fired more than three times during the exploration phase; 2) when computing the mutual information between its Ca^2+^ events and the animal’s position, the correlation was greater than the 99th percentile of the spatial information calculated for the shuffled dataset; 3) there was no evidence of spike burstsl and 4) a minimum number of entries in the place field (*Materials and Methods* contains details). With these criteria, about a third of active cells were classified as PCs (29 ± 5%; *SI Appendix*, Fig. S7*D*), which is in line with previous reports in various environments by using different recording techniques (23, 32–35). Overall, 59 ± 8% of cells was classified as a PC in at least one session, which is in agreement with ref. 23 (*SI Appendix*, Fig. S7*E*). The majority of PCs had one place field, which is in agreement with previous reports (23, 36, 37) (*SI Appendix*, Fig. S7*F*). An exact number is likely influenced by the extent of effective behavioral sampling of the arena and the stringency of the classification criteria.

The next step was to examine whether spatial information in the activity of recorded neurons was sufficient to correctly predict the animal position. Using the data from the 10-min daily exploration phases (Fig. 2*E* and *SI Appendix*, Fig. S8), we trained a decoder to predict the animal position when at one of the three empty sandwell locations. Fig. 2 *E*, *Right*, shows that the probability of decoding the correct location of the animal (data) was significantly above chance performance (shuffle). Thus, the decoder correctly predicted the location of the sandwells from the unit activity (i.e., that the animal was located there; t values = 2.96, 4.56, and 3.01 for sandwell 1, 2, and 3, respectively; data vs. shuffle, df 8, all *P* < 0.02). As expected, most of the spatial information was contained in what were classified as PCs; with a decoder using only the activity from PCs, the performance was similar to the performance using all cells (all cells vs. PCs, df 8, *P* = 0.43, one-way ANOVA multiple comparisons) and above chance (data vs. shuffle, *P* < 0.001 in both cases, one-way ANOVA multiple comparisons; Fig. 2 *E*, *Center*, in the second row). In comparing the performance of all cells with those of defined PCs, we also observed a trend for some residual spatial information to be contained in this other subpopulation of cells. However, among this group of cells, only some may be contributing to performance (other cell data vs. shuffle, *P* = 0.083, one-way ANOVA multiple comparisons, nonsignificant).

The next issue was to examine if neurons were reliably reactivated by traversing analog portions of space during repeated outbound and then inbound trajectories between the startbox and the rewarded sandwell during sample and choice trials. The pairwise cosine dissimilarity was computed between the frames in accurately directed outbound paths of sample trials (i.e., the animal reached the rewarded sandwell first) and correct choice trials. Dissimilarity was lower in time points of corresponding positions, as presented in Fig. 2*F*. The repeated pattern of low cosine dissimilarity parallel to the diagonal indicates that as animals traversed the same space, the activity of neurons in the sample and choice phases was also similar. This repetition can be observed by expressing their distance with a multidimensional scaling (MDS) manifold representation (Fig. 2*G*). Each point on the manifold represents the neural population vector at a particular time point of the outbound path, and the distance between points represents the relative cosine dissimilarity. For both sample and choice trials, the intrinsic dimensionality (38, 39), a measure of the number of dimensions required to describe the neural activity, was on average 1.91 ± 0.36 dimensions. Indeed, the first two components contained most of the spatial information (Fig. 2 *G*, *C1-C2 Inset*; *SI Appendix*, Fig. S9), while additional dimensions typically included trial-to-trial variations such as whether a trial was correct or incorrect (*SI Appendix*, Fig. S9*A*). The manifolds of the individual trials displayed higher than chance correlation, especially in the first two components (*SI Appendix*, Fig. S9*D*), indicating that repeated traversals recruit similar activity along their length. This likely represents, in part, the population of PCs along the trajectory, but interestingly, the start of the manifold trials (the last 10 s in the startbox) was close to the end-point in 2D space (i.e., arrival at the rewarded sandwell), and the manifold assumes a circular shape, especially in the C1-C2 plane (Fig. 1 *G*, *Right*, and *SI Appendix*, Fig. S10). As the closer two points are to each other in manifold space, the lower is their cosine dissimilarity; these data point to the striking finding that the population neural activity in the startbox is similar to that of the correct sandwell.

### Neuronal Activity in the Startbox Is Predictive of Spatial Decision-Making.

**Fig. 3.**
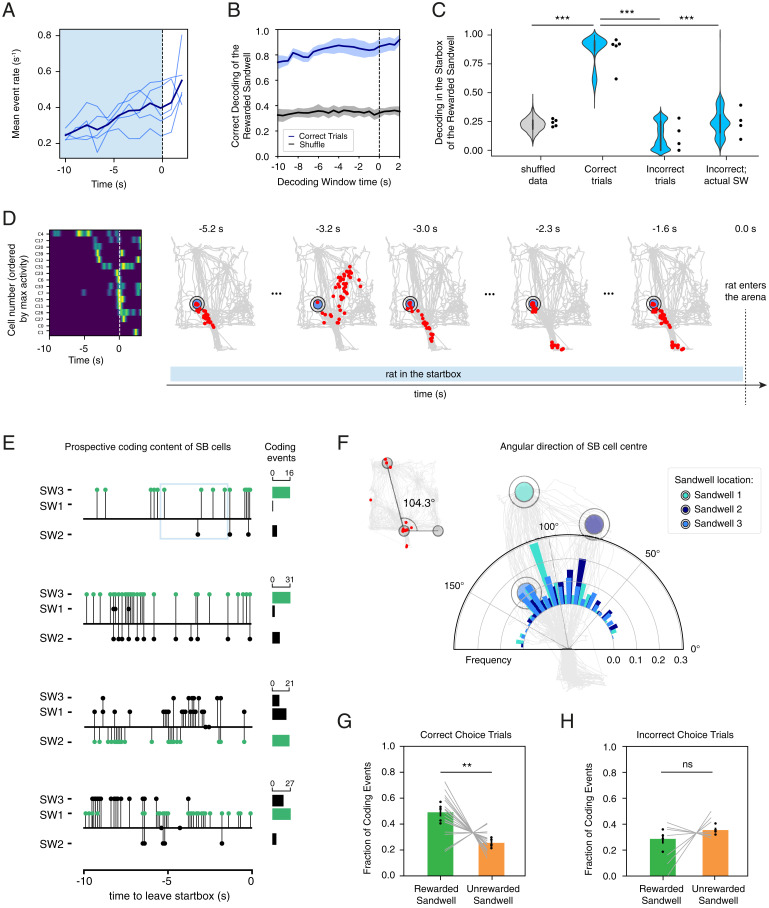
Prospective coding during decision-making. (*A*) Neural activity increased as the moment of leaving the startbox approaches (dashed line). Mean event rate (events per second) in the 10 s before leaving (blue shaded area). Individual animals are shown in light blue and the population average in blue. (*B*) The success of decoding of the correct destination sandwell increases as the moment of leaving the startbox approaches (dashed line). Mean ± SEM is resented in blue and shaded area, while the mean ± SEM for the control shuffled data are represented in gray and shaded area. (*C*) Decoding the performance of the destination in choice trials from the startbox activity. The performance was highly accurate in correct trials compared to the shuffled data. In incorrect trials, instead, the performance was much worse and at chance level. For incorrect trials, the decoder performance was inaccurate also when they were relabeled with the first visited sandwell (incorrect actual) during those trials. ***P < 0.0001, one-way ANOVA comparison of means. (*D*) A representative example of cell events during the 0 s before leaving the startbox and the first 2 s of the trial in the arena. Cells are ordered by maximum activity. On the *Right*, we plot the spatial content of the cell activity between −5.2 s and −1.6 s. Red dots are the position of the events when the cells were active in our experiment (which includes the arena and the startbox). Importantly, during the display window, the animal was in the startbox. (*E*) Representative examples of choice trials showing the repeated prospective coding of the different sandwells (SW) in the 10 s before leaving the startbox. Prospective coding corresponding to the correct sandwell in each trial is displayed in green. Next to each graph, the frequency of each prospective coding is displayed as a histogram. The first trial on *Top* corresponds to the trial displayed in *D*; the light-blue square indicated the portion of the trial presented for display purposes. (*F*) Histogram of the angular distribution of the place centers for the cells active in the startbox divided by the identity of the rewarded sandwell (1, 2, or 3). Individual histograms are presented in *SI Appendix*, Fig. S17. (*G*) Relative number of prospective coding events for the rewarded and nonrewarded sandwells during correct trials. Black dots are individual animals and gray lines are individual sessions. **P = 0.0057, paired *t* test. (*H*) Number of prospective coding for incorrect trials. ns = 0.3033 paired *t* test.

We have seen that, over sessions 18 to 21, the animals chose the sandwell to approach upon leaving the startbox in choice tests at around 90% correct. The manifold results presented above prompted us to gather further insight into this initial part of the trial and, in particular, the last 10 s in the startbox before leaving. Note that the animal has full agency about when to leave after the door had opened. Fig. 3*A* shows that mean event rate rose steadily during this 10-s period. A decoder was then trained on the activity in the startbox in 2-s time windows (Fig. 3*B*) to see if it would correctly classify to which sandwell the animal was headed during choice trials. The prediction accuracy rose to >80% as the moment of entrance into the arena approached and was significantly higher than chance when compared to shuffled data that were, as expected for 3 sandwells, around 33% (Fig. 3*C*, breakdown of individual animals is presented in *SI Appendix*, Fig. S11). The decoder correctly identified the sandwell to which the animal was heading before leaving the startbox on trials when the animal chose correctly but poorly when the animals’ choices were incorrect (i.e., they searched at a nonrewarded sandwell first in that trial). This prediction was highly significant for correct trials (ANOVA, F = 43.84, df 3/14, *P* < 0.0001).

Interestingly, the prediction was not significant for incorrect trials (*P* = 0.46 to shuffled data, one-way ANOVA multiple comparisons). Decoding performance remained poor when we reclassified the incorrect trials as leading to the appropriate sandwell (but a sandwell that was nonrewarded on that session) (*P* < 0.0001 compared to correct trials, *P* = 0.49 compared to incorrect trials, one-way ANOVA multiple comparisons). Together, these data indicate that startbox neural activity is not anticipating a different sandwell during incorrect choices but rather is not discerning between possible choices at all; the resulting outcome is perhaps a casual behavioral choice. However, if random, this choice may sometimes lead to the correct sandwells, and indeed, we noticed a small proportion of correct choice trials where decoding performance was poorer (Fig. 3*C*). A trend for better decoding performance on correct trials as a function of the number of cells active in the startbox was observed, but overall performance was higher than 65% (*SI Appendix*, Fig. S12). Interestingly, a lower but significant performance was observed in later sample trials when the animal went to the appropriate sandwell directly, indicating that even a small number of prior exposures may be sufficient to drive memory encoding and a coherent representation during retrieval (*SI Appendix*, Fig. S13 *A* and *B*). This is unlikely to simply reflect the direction the animals were facing because we could not predict the animals’ goal from the orientation of their head (*SI Appendix*, Fig. S14).

A high proportion of the cells active in the startbox was reactivated when the animal was in the arena (t = 5.98, df 4, *P* = 0.0039, paired Student’s *t* test compared to cells active in the startbox only; *SI Appendix*, Fig. S15 *A* and *B*). In particular, startbox events were significantly more likely to map to the portion of the arena occupied during sample and choice phases, reflecting the paths taken to and from one of the three sandwells (t = 3.459, df = 4, *P* = 0.026, paired Student’s *t* test; *SI Appendix*, Fig. S15 *C* and *D*). Moreover, the majority of startbox cells (64 ± 12.5%, mean ± SEM) represented the animals’ outbound paths (with ∼22% PCs). Conversely, only 3.6 ± 0.2% (mean ± SEM) were active during the inbounds path (of which ∼6.5% were PCs) but 39 ± 6% (mean ± SEM) were active in both inbound and outbound paths (*SI Appendix*, Fig. S16). To determine the information content at each time point during the 10 s before leaving the startbox, the locations of place fields in the arena were plotted for the subset of cells active in the startbox during this time window (displayed in Fig. 3*D* is the time window from −5.2 s through to −1.6 s). Fig. 3*D* shows exemplar activity patterns at different time points before the animal left the startbox on an individual trial at specific time points. This was a trial in which the animal went to sandwell 3. The initial activity corresponds to the correct sandwell and the trajectory leading to it and then a momentary change of anticipation switching to sandwell 2 (at −3.2 s) followed by a return to the commitment to approach sandwell 3. Note that the rat was in the startbox throughout this period (*SI Appendix*, Fig. S14*F*). This exemplar pattern was quite common across different animals and sandwells, and we therefore refer to this phenomenon as “prospective coding,” as the temporal resolution of calcium imaging is insufficient to use the term replay (*Discussion*). In Fig. 3*E* and *SI Appendix*, Figs. S17 and S18, further examples are displayed showing that different patterns of prospective coding occur over time, corresponding to the correct sandwell location or correct trajectory (in green) but also of the other two sandwells (black).

The direction to which startbox cells were pointing was quantified as the average angle between the spatial location of the cell events during exploration and the door of the startbox (Fig. 3*F*). As expected, the distribution of startbox directions corresponded to the angles of the directions to the three sandwells (Fig. 3 *F*, *Inset*). This distribution changed between sessions due to the use of different rewarded sandwells in a random but counterbalanced order (Kolmogorov-Smirnov comparisons between sandwells 1, 2, and 3: all D values are >0.19 with all *P* values of <0.0001). In particular, the distribution peaked toward the angle corresponding to the correct sandwell of that session (Fig. 3*F* and *SI Appendix*, Fig. S19). The number of prospective coding events in the startbox was strikingly higher on correct than incorrect trials (Fig. 3 *G* and *H*; paired Student’s *t* test, repeated measures, t = 5.41, df = 4, *P* = 0.0077). Note that the number of prospective coding events associated with the other sandwells was not zero—consistent with the pattern jumping around during the startbox decision-making period. On incorrect trials, the number of prospective coding events was similar for the correct and incorrect sandwells (paired Student’s *t* test, repeated measures, t = 1.239, df = 3, *P* = 0.3023). On average, prospective coding events corresponding to the recall of the correct sandwell in the startbox were longer and recruited a larger number of cells than those of the incorrect locations (paired Student’s *t* test, repeated measures, t = 4.372 df = 4, *P* = 0.0119) (*SI Appendix*, Fig. S20 *A* and *B*). Furthermore, the number of events for the correct sandwell was relatively constant on all 6 trials in the choice phase (*SI Appendix*, Fig. S20*C*). However, this was markedly different for sample trials during which the animal has to encode the new daily location, for which the first three trials are closer to chance, while the last three show more frequent prospective coding of the correct sandwell (*SI Appendix*, Fig. S20*C*).

## Discussion

There are four main findings. First, calcium imaging of large numbers of cells in freely navigating rats in an open arena has confirmed years of electrophysiological data showing that PCs and other cells with spatial selectivity can be observed, with no global remapping of place field locations observed between exploration, sample, and choice phases. Second, neural activity in the startbox increases during the waiting period before a trial starts with population coding activity preferentially correlating with any correct target or path to which the animal is about to run. Third, startbox activity is not predictive of the goal on trials when the animal performs incorrectly. Fourth, population coding of cell activity reveals that the activity in this time period prospectively codes the trajectories to possible goal locations, with the correct goal location being overrepresented. These data collectively reveal that neuronal activity corresponding to possible destinations or the paths to them is reactivated in the time period before a decision is made, which is a pattern of neural activity that could inform decision-making by displaying alternative scenarios.

These observations were possible through the successful optical recording with miniscopes in the CA1 hippocampal region of rats performing a complex, naturalistic navigational task in a large environment. The application of this technique to the rat required the careful aspiration of a very small strip of myelinated alvear fibers overlying the hippocampus, as well as those of the corpus callosum (as used in the mouse; *SI Appendix*, *Supplementary Discussion*), in such a manner as to leave stratum oriens undamaged (Fig. 1). In this way, there was minimal damage to the hippocampus itself, CA1 cell imaging was feasible, and we realized successful, reliable recordings over many days with stable regions of interest. Rats typically exhibit a wider range of behaviors and are better suited for demanding neuroscience protocols such as those employed in the study of spatial and episodic memory (40–42). Compared to electrophysiology, calcium imaging allows the identification of neurons across different sessions and for prolonged periods of time (23), although at the expense of time resolution and sensitivity. Ref. 37 also provides a detailed comparison of data from the rat and mouse, and our joint introduction of the technique to one of the most studied areas of the brain in one of the main model organisms is particularly important.

Our first finding confirms that this technique is effective in the detection of PCs in the rat. With our criteria (*Materials and Methods*; 99% mutual information), a third of cells were classified as PCs, with the remaining population of cells also exhibiting some degree of spatial selectivity (Fig. 2*E*). Different reports estimate PCs to range from 20% (43) to 35% (35) to 80% (32), although the number is markedly influenced by task factors, which are the criteria used to define PCs and which cells can be included in population encoding. Our results are broadly in line with reports on rat hippocampus using electrophysiology in 2D environments, despite certain parameters (such as rate of firing) being different in optical recording. Shifts between environments (2, 44, 45) or prominent changes in environmental cues (46) are known to induce global, partial, or rate remapping of PCs. Here, we did not observe global remapping between exploration, sample, and choice trials, although we cannot exclude some level of rate remapping in the firing rate of PCs between the different phases. Indeed, in related experiments, it has been reported that even the introduction of barriers in a well-known environment can allow stable place fields to be maintained (16). With receptive fields stable across days, this greatly expanded the neuronal dataset with which to assess navigational trials and train a decoder. In our experiment, place fields remained largely stable across days (Fig. 2 *C* and *D*) as also reported in ref. 27. This stability is likely to be due to the animals’ extensive training in the same arena. While large day-to-day variability has been reported between early epochs of an environment (23), correlation in the neural representation between sessions increased as the animals became acquainted with the environment upon repeated presentations (25).

Second, before the animals take any action to move toward one of the sandwells, neural activity increases in frequency such that, by the time animal chooses to leave the startbox, there is enough information in population measures of activity to correctly predict the animal’s destination, at least for correct trials (Fig. 3 *B* and *C*). Interestingly, similar measures on the first or second sample trial, before the animals had fully encoded which is the correct location that day, were poorer, but activity thereafter assumed a predictive pattern (*SI Appendix*, Fig. S20*C*). Thus, spatial recency within a well learned context can be encoded and accessed remarkably fast, which is reflected also in the reduced number of behavioral errors after the initial sample trials (Fig. 1*J* and *SI Appendix*, Fig. S5). In at least a fraction of cells (65 ± 3%), the information contained in the startbox neuronal activity reflects spatial information about one of the sandwell locations or the outbound trajectory to reach it (*SI Appendix*, Figs. S15 and S16 and Fig. 3 *D*–*H*). These observations are consistent with a report by Komorowski et al. (47) that showed that PCs may become engaged by the task on which animals are trained such as the spatial recency task here .

Third, we observed that startbox activity was different on behaviorally incorrect trials. The relatively higher representation of the correct sandwells likely reflects a focus on the imminent destination (Fig. 3 *E*–*G*). At the beginning of incorrect trials, and in early sample trials, the proportions of prospective coding events for the correct and incorrect sandwells were statistically indistinguishable (Fig. 3*H*). This finding is consistent with the results obtained using the decoder (Fig. 3*C*) where neither the correct nor the actual destination could be decoded from the neural activity in the startbox on incorrect trials.

Fourth, population coding enabled the analysis of activity patterns using state-of-the-art, multidimensional manifolds to represent neural population activity in multidimensional spaces. It was observed that two dimensions were sufficient to capture most of the variance of the cosine dissimilarity in the neural activity recorded in the animals’ trajectories to the sandwell. A striking observation was that, on the outbound path of correct choice trials, these visualizations assumed a circular appearance suggesting a relationship between the population firing in the startbox and at the correct sandwell. This was confirmed by observing the spatial content contained in the activity of the population of cells active in the startbox. Such representation cannot be measured using only single cells and reflects a potential strength of calcium imaging. Compared to electrophysiology, calcium imaging allows the identification of large numbers of neurons across different sessions and for prolonged periods of time (23, 24), although at the expense of time resolution and sensitivity. While replay has been traditionally recorded by means of electrophysiology because of its good temporal resolution, there have been reports of replay with calcium imaging (21, 22). Although it is likely that replay detected with electrophysiology and cell reactivation detected in this work represent the same biological phenomenon, we deemed it prudent to refer to our finding as prospective coding.

These observations of spatially predictive activity collectively support findings that suggest that prospective coding could be used to guide future behavior (48, 49). While some authors have proposed this, other reports have argued that replay may rather reflect past memory, as past trajectories seem to be overrepresented in reward-switch tasks (19). Here, we find that prospective coding not only maps onto future, correct trajectories but also includes momentary mapping onto the other sandwells during decision-making in the startbox (Fig. 3 *D*–*H*). Our proposal is that such activity can sometimes serve to anticipate possible scenarios during decision-making. Accordingly, different outcomes may be weighed during decision-making to guide correct goal-directed behavior.

The behavioral task used, now referred to as everyday memory, has the defining characteristic of the episodic encoding of information (e.g., during nondiscrimination sample trials) that can be accessed for a period but is typically forgotten quickly—a common everyday experience. By session 18, the animals were completely familiar with the spatial context and likely had a consolidated representation in long-term memory of the environment. Their task was to update each day which area of space mattered—spatial recency. The free choice and the flexibility of this task are extra factors to encourage the animal to think about the desired destination. When the choice is more forced by the configuration of the task, as in the case of radial mazes (19), the replay of past trajectories may be preferred to avoid choosing a wrong arm that precludes access to the rewards by not allowing rerouting, in analogy to what has been reported in experiments with fear conditioning (17). Indeed, the data by Gillespie et al. (19) show that, after repeated trials, the representation of the rewarded arm increases. Our data therefore support a role for prospective coding to inform decision-making rather than only guide future behavior, as also theoretically suggested by others (50, 51). Prospective coding by hippocampal CA1 cells may hence prioritize paths that are most immediately relevant for on-going behavior (52). This would also explain how prospective coding can even construct mental scenarios that have never actually been experienced (11). It has been reported that differential activity along common paths can reflect a future choice (8, 9). However, most cells we identified in the startbox were also active in the arena at some point; it is possible that the different configuration of our behavioral setup does not promote differential firing because there are no obliged common tracks reaching the various sandwells. Indeed, cells only active in the startbox were a smaller proportion of the total (*SI Appendix*, Fig. S15*A*), and their activity may be differentially regulated by the animal’s destination (9). An alternative possibility is that via the prospective coding of different trajectories, the brain is performing evidence accumulation until some threshold is reached, possibly with conjunctive coding by other brain regions, e.g., in anticipation of a reward (53). Indeed, Fig. 3*E* suggests that as the decision point approaches, the number of prospective coding events for the correct sandwell increase. Future work will elucidate this aspect.

There are limitations to our observations. One is that, due to technical reasons, it is difficult to reconstruct sequences of recorded neurons or to ascertain their ordered activity below the frame-rate detection of 20 Hz. Accordingly, we use the neutral term of content of neural activity rather than considering them as sequences. One additional caveat is sensitivity, such that realistically isolated action potentials are not detected by GCamp6f. This likely results in an underestimation of the number of prospective coding events and of the number of cells involved. Nonetheless, we find that a third of cells active in the startbox at the beginning of trials are common in any other trial (*SI Appendix*, Fig. S13 *C–E*). This should affect all activity in an equal manner, so we doubt it introduces significant bias to our analysis; if anything, our results provide a conservative estimate of the frequency of such prospective coding events.

In conclusion, we have presented an application of miniscope imaging of the rat CA1 to the study of decision-making in spatial navigation. This offered the possibility of comparing the neuronal activity across multiple sessions in which animals encode the most recent rewarded location and engage in memory prospective coding to go to it. Before any action is taken to navigate toward one of the possible destinations, the activity corresponding to three possible locations or trajectories is prospectively coded by hippocampal neurons. Our results suggest that such coding may be used by the animals to recall alternative destinations and, possibly, to evaluate their suitability. Our data provide information regarding the role of prospective coding during remembering/action planning. Future directions will further clarify how the animals use this information to plan and simulate possible paths and how the choice is made from these possibilities. Furthermore, it will be interesting to test how allocentric or egocentric spatial features of the task influences prospective coding.

## Materials and Methods

### Animal Subjects.

Lister-Hooded rats (*n* = 8) were 2 to 3 mo old at the start of the experiments. Animals were purchased from Charles River and group housed until surgery; after surgery, animals were housed in single cages. During the behavioral task, they were food deprived to 85 to 90% of the free-feeding weight against a growth curve, with free access to water, on a 12:12-h light-dark cycle with training in the light phase. Care of the animals complied with the UK Animals (Scientific Procedures) Act conducted under a Project License (PPL P7AA53C3F). Two animals were excluded from the recordings due to insufficient quality of the field of view (FOV; low number of detected cells). One further animal (H0487) could only be recorded in sessions 18 and 19 due to technical issues and was excluded from the analysis on prospective coding because we could not compare the three possible destinations.

### Immunohistochemistry.

At the end of the experiments, the animals were anesthetized with 200 mg/mL pentobarbital and perfused transcardially with cold PBS (phosphate buffer saline; P4417 Sigma Aldrich) and 4% formaldehyde in PBS (Sigma Aldrich 441244). Heads were postfixed for 24 h in formaldehyde, and then the brains were extracted and postfixed overnight. They were transferred in 20% sucrose PBS and cut with a cryostat (Bright Instruments). After being washed in PBS, slices were permeabilized in PBS supplemented with 10% NDS (normal donkey serum; Sigma Aldrich D9663) and 0.1% Triton X-100 (Sigma Aldrich T8787) for 30 min, and then incubated in PBS supplemented with 10% NDS 0.1%, Triton X-100, mouse anti-GAD67 antibody (Sigma Aldrich MAB5406 clone 1G10.2) 1:1,000, and guinea pig Anti-NeuN (SYSY 266 004) 1:500 overnight at room temperature. The next day, three washes with PBS with 0.1% Triton X-100 were performed, and then slices were incubated in PBS with 0.1% Triton, 1:200 donkey anti-mouse Alexa 647 (Thermo Fisher A-31571) and 1:200 anti-guinea pig Alexa 555. After three washes in PBS, the slices were mounted in Fluoroshield with DAPI (Sigma Aldrich F6057).

### Microscopy.

Multichannel images were acquired using a Nikon A1R Ti:E inverted confocal microscope with 1AU pinhole dimension, using a 40× Plan Apo/numerical aperture (NA) 1.25 oil objective (histological staining) or 10× Plan Flour/NA 0.3 (GCaMP6f/DAPI acquisition). Sequential acquisition of the four channels was performed with the laser lines DAPI 402 nm, GCaMP6f 488 nm, NeuN/Alexa555 562 nm, GAD67/Alexa647 639 nm, and 450/50; and with 525/50 and 595/50 filter cubes. Histological sections stained with DAPI were images with a Leica DMR upright microscope with Retiga 2000R camera using an epifluorescence mercury lamp and PL FLUOTAR 10×/0.30 and PL FLUOTAR 20×/0.50 objectives.

### Surgical Procedures.

Surgical procedures were performed under sterile conditions and according to best practice. Animals were maintained under 3 to 2% isoflurane (Covertus absis01) reversible anesthesia and 0.5 mL rimadyl (Zoetis) administered as an analgesic at the beginning of each surgery. In the first surgery, 1 µL of AAV1.CaMKII.GCaMP6f.WPRE.SV40 (UPenn Vector Core, then Addgene 100834-AAV1) diluted with sterile PBS (Sigma-Aldrich) to a final concentration of 5.7 10^12^ vg/mL was injected in the CA1 region of each hemisphere (stereotaxic coordinates from bregma AP (Antero-Posterior) 3.6, ML (Medio-Lateral) ± 2.2, DV (Dorso-Ventral) 2.2 from dura) at 100 nL/min using an automated injection pump and a Hamilton syringe equipped with a Nanofil needle (World Precision Instruments). The GRIN lens was implanted 7 to 10 d after the first surgery. In the second surgery, three surgical screws were implanted in the skull (Screws and More, DIN 84 A2 M1 × 3). A circular craniotomy was performed with a trephine for microdrill (Fine Science Tools 18004-18) at AP 3.8, ML 2.4. A cylindrical volume of cortical tissue was then aspirated manually with a sterile blunt needle in the 27G to 30G range. Constant irrigation with cold saline is performed during aspiration to prevent swelling and clean blood. A small portion of alveolar (anteroposterior) and callosal (coronal) fibers was then removed with the same blunt needle with mild aspiration to expose but leave undamaged the outer surface of the hippocampus (corresponding to CA1 stratum oriens). Cold saline irrigation and soaked sterile gelatin sponge (we successfully used Pfizer Gelfoam or Delta Surgical [Newcastle Under Lyme] Gelita-Spon GS-110) were used to keep the hole clean until complete stop of the bleeding. A 1-mm-wide, 9-mm-long GRIN lens (Inscopix) was then lowered with a 5-degree angle in position AP 3.8, ML 2.4, DV 1.9 from dura (the dura is measured before the aspiration). Two stainless-steel rods (0.09-mm diameter; CrazyWire UK) are attached to side of the GRIN lens with superglue before implantation, with around 3 mm of rods protruding from the surface of the GRIN lens. A thin layer of surgical silicone (KWIK-SIL, World Precision Instruments) was applied to the sides of GRIN lens to prevent cement to enter in contact with the brain tissue. The GRIN lens was cemented in place with Super Bond dental cement (Prestige Dental) to cover the skull and included the skull screws.

### Calcium Recordings.

The Inscopix nVista system was used to perform calcium imaging experiments. At least 3 weeks after the GRIN lens implantations, the animals were anesthetized temporarily and the FOV checked with the Inscopix miniscope. The baseplate support for the miniscope was scored with a scalpel blade to provide better attachment with SuperBond cement. With the miniscope, the optimal position for the baseplate above the skull was determined by looking for the FOV containing neurons. Some brighter neurons are usually seen along with capillaries, and some neural activity could be sometimes detected by very briefly lowering the anesthesia depth to 0.5 to 1% isoflurane before raising it again. The baseplate was then cemented in place with SuperBond using the pre-existing layer of cement. Scoring the previous cement surface with a scalpel blade is recommended to ensure optimal adhesion. After the cement had hardened, the miniscope could be removed with the baseplate now anchored to the animal skull. A small piece of duct tape can be used to prevent dirt and dust to enter the cavity between the baseplate and the lens while not recording.

When recording, the miniscope is positioned on the baseplate and secured with a miniature screw attached to the baseplate, taking care that the animal movement does not displace the miniscope from the correct position. Proper handling of the animals is fundamental in our experience, and extensive habituation is required so that the miniscope can be attached to the animals’ head without the need to completely immobilize them or use anesthesia. Rather, a mild restraint of the animal’s head at jaw level, without causing distress, is often enough to enable the positioning of the miniscope. The miniscope cable was connected to a commutator on the ceiling that enables full animal rotation. The commutator was connected to the nVista imaging system (v 3.0) that controls the miniscope functioning and stores the recorded data. Because we were recording from a large 2D area (1 to 2 m wide), the cable should be long enough to allow easy access to the whole arena. To prevent the cable being uncomfortable, elastic wires were used to connect the two extremities of the cable. On the first day of recording, the exact FOV was chosen by fine tuning the eFocus parameter on the miniscope that controls the focus of the internal lens.

Ca^2+^ recordings were collected at 20 Hz for a 1,061 × 800-pixel FOV using the Inscopix nVista imaging system (v3.0) and synchronized with the camera behavior via an electronic signal in one of the GPIO channels timestamping the time of a small light-emitting diode (LED) light in the camera FOV at the beginning and end of the recording. The camera has a pixel size of 0.82 μm.

### Apparatus.

All experiments were conducted using a modified event arena (29) that was 1-m × 1-m square. The walls (40 cm high) are transparent, and the floor is composed of 20-cm × 20-cm removable white tiles (for regular cleaning purposes). Three Plexiglas sandwells (6-cm diameter, 4-cm depth) that contained the hidden reward pellets were placed in one or a subset of the panels with holes. To mask the smell of the food, the sandwells were filled with clean rodent bedding material. Each sandwell had a spherical plastic bowl that enabled rewarded sandwells to contain one or more reward pellets (BioServ F0171) (0.5 g). Nonrewarded sandwells contained an equal number of reward pellets in a space underneath, thereby serving as inaccessible food. These plastic bowls had holes ensuring that the rewarded and nonrewarded sandwells contained the same number of reward pellets at approximately the same depth in the sand and thus should exude the same smell. Extensive randomizing and counterbalancing were also arranged to minimize olfactory artifacts, as follows: 1) the sandwells used in the encoding trial were not used for the recall trial of the same session; 2) all sandwells were used as rewarded or nonrewarded sandwell across days; and 2) the arena floor was regularly wiped with a 70% alcohol-impregnated towel between sessions, and before recall and probe trials. Animals entered the arena from a single startbox located nominally in the south. Two intramaze cues were positioned inside the arena on the west and east walls, namely, a multicolor toy and a clean yellow mustard dispenser, respectively. Two prominent extramaze cues were present outside the arena.

### Habituation.

Rats were first taught to dig for food in sandwells inside their home cages. In a first habituation session in the arena, the rats were permitted to explore the arena with two intra-arena cues and surrounding extra-arena cues for 10 min. They were also habituated to being put in a startbox and given a 0.5-g food pellet to eat. When the pellet was eaten (typically around 30 s), the rats were allowed 10 min access to the arena. On the second and third habituation, one 0.5-g pellet was placed on top of the sandwell; rats collected the pellet and took it back to the startbox; rats were then allowed to retrieve other food pellets buried close to the surface of the sandwell. If rats had difficulties in retrieving the hidden food pellets, they were helped by exposing one further pellet in front of them.

### Behavior.

Rats were trained in a modified form of the event arena task (29). In this task, animals learn to retrieve food pellets from one of the three sandwells in the everyday arena, whose position changes from one session to the next (sandwell 1, 2, or 3). The task is divided in three parts. During exploration. animals explore the arena for 10 min without any reward present and the sandwells are empty. During encoding (sample phase) one sandwell chosen at random is rewarded and contains 0.5-g food pellets. Animals are trained to retrieve six pellets from the sandwell, entering the arena from the startbox after the door opens, retrieve one pellet from the sandwell, and return to the startbox to consume it. During the choice trial, all sandwells were filled with bedding and appeared identical on the outside. The correct sandwell had pellets in the accessible bowl, while the other sandwells had their equal number of pellets in the nonaccessible compartment. The choice trial was performed 30 min after the end of the sample trial. During the choice phase, the animals retrieved six pellets, returning to the startbox to eat each one. A 0.5 s, a 2,300-Hz tone was played 5 s before the door opened.

### Data Analysis.

#### Behavior performance.

For each trial, latency and the number of errors were quantified. Latency is the time that occurred between the moment that the animal leaves the startbox to when it starts digging at the correct sandwell. The number of errors is the number of incorrect sandwells the animal dug at before reaching the correct sandwell, and in this experiment, we can assume values 0 (the correct sandwell is the first choice) or 1 or 2 (the animal dug at all sandwells before reaching the correct one). The performance was defined as

[1] $$\mathrm{Performance}=1-\frac{n}{n_{\max}},$$

where *n* is the number of errors. Hence, if the animal performs at chance it results in Performance = 0.5, as shown in Eq. 2.

[2] $$E\left(n\right)=\sum_{i}n_{i}P(n_{i})=0\left(¼\right)+1\left(½\right)+2\left(¼\right)=1$$

#### Single-photon calcium imaging.

For each subsequent Ca^2+^ recording, the Inscopix data processing software (IDPS; Inscopix v1.6) environment was used to denoise the recording by applying a low and high filter spatial bandpass (σ _low_ = 0.0005 and σ _high_ = 0.5). IDPS was then used to correct motion artifacts by using a rigid motion correction algorithm (54, 55). Preprocessed recordings were then imported into the CNMF-E python application programming interface (API) (56), their cell ROIs identified and their respective calcium transients computed as the change in fluorescence over baseline fluorescence as ΔF/F = (F − F_0_)/F_0_, where F_0_ is the mean fluoresce over the trace. All ROIs and their respective traces were manually inspected, with any duplicates or artifacts discarded. Neural events were then computed from the calcium traces using the Online Active Set method to Infer Spikes (OASIS) package (57, 58). For each animal, calcium traces were manually assessed for levels of noise and a unique noise threshold applied (typically an “s_min” parameter of between 0.2 and 0.3 was used). Longitudinal registration was performed in the IDPS. Individual recordings from the same sessions were first aligned generating one dataset for each session (S18 to S21) and then the four datasets were aligned together. Correct alignment was verified by screening a random subset of an individual set from the correspondence table generated by the system.

#### Animal positional tracking.

Behavioral recordings of the task are performed with a camera placed on the ceiling (black and white, 20 fps). A small LED light outside the arena was used for the alignment and switched on briefly while an electrical signal was sent to one of the nVista GPIO channels. The ON time can be easily detected by a change in intensity in the corresponding pixels. Rat positional trajectories were tracked using the Python image recognition deep convolutional neural network Deeplabcut (DLC) (59).

#### Training dataset.

A training set of 180 distinct frames of the animals in the arena in all stages (60 frames from 3 rats) were extracted based on *k*-means derived quantization. This involved downsampling the video and modeling individual frames as vectors and then randomly selecting frames from different clusters. Each frame in the dataset was then manually labeled using the point between the animal’s ears as reference.

#### Network training.

A 50-layer deep pretrained convolutional neural network (ResNet-50) was then refined by training it for 500,000 iterations on the training dataset and evaluated using the mean average Euclidean error between manually labeled frames and those predicted by DLC. Accuracy was also manually checked and corrected by referring to a DLC-labeled video. The position of the animal is translated using one corner of the arena as the reference origin.

#### PC identification.

Spatial tuning was inferred using custom Python scripts. For each neuron, the Kraskov Spatial Information (SI) was calculated between the binarized event train and the vector of the animals binned spatial activity (obtained by binning the arena into 4- × 4-cm spatial bins). A neuron was classified as a PC if it met the following criteria: 1) the neuron had at least three events during the imaging stage, 2) the animal traversed bins in which the neuron was active at least three times, 3) a neural event occurred in at least 20% of traversals, and 4) the neurons’ SI exceeded the 99th percentile of a distribution of SI for 5,000 shuffled neural event trains.

#### Place fields.

A neuron’s place field was defined by calculating its rate map and normalizing by the animal’s occupancy map and then selecting all contiguous bins that surrounded the place field center (i.e., the bin with maximum activity). Place field size was then calculated as the number of 4-cm bins squared in the place field.

#### Decoders.

A Gaussian naive Bayes (GNB) python algorithm from Sckit-learn was used for sandwell classification using neural activity first within the arena and subsequently from within the startbox. In both cases, neural event trains were convolved with a Gaussian kernel (σ = 200 ms, window width of 4σ) to obtain a time series of instantaneous event rates.

#### Sandwell classification arena.

For sandwell classification within the arena, sandwell regions were first defined as the area within a 5-cm radius of the well center. All exploration sessions were concatenated and sandwell activity isolated to produce an N × T matrix of instantaneous event rates (*X*), where N is the number of neurons that are active across all sessions and at least one sandwell and T is the amount of time the animal spent in the sandwell. Then *X* was labeled with the animal’s corresponding sandwell (y) at each point in time. The number of observations for each sandwell class was balanced by downsampling the number of samples for each well to that of the least visited and repeated 10 times to enable random sampling of the sandwell activity. Finally, a 10-fold cross-validation strategy was conducted with a 70:30 train: test split. For each training set, GNB assumes that the class-conditional densities, P (*x_i_* | y), are normally distributed (as shown in Eq. 3, with *µ_y_* and *σ_y_* the mean population vector and stanard deviation for class *y*, respectively). Finally, the Maximum A Posteriori estimation (as shown in Eq. 4) was used for each sample, yielding a predicted sandwell for each point in time:

[3] $$\mathbb{P}\left(x_{i}\;|\;y\right)=\frac{1}{\sqrt{2\pi \sigma^{2}}}\exp\left(-\frac{{\left(x_{i}-\mu_{y}\right)}^{2}}{2\sigma_{y}^{2}}\right),$$

and

[4] $$\hat{y}=\text{argmax}\;\mathbb{P}\left(y\right)\prod_{i=1}^{n}\mathbb{P}\left(x_{i}\;|\;y\right).$$

#### SB classification of sandwell.

Using correct sample and choice trials, a GNB decoder was trained to identify the animal’s prospective sandwells (the sandwell that the animal was about to visit). For each trial (six sample, six choice), the startbox activity was isolated as the 10-s period before the animal left the startbox and entered the arena. Trial activity was merged into a single matrix (Trial × N × T_SB_) and temporally split into 5 × 2-s windows, starting with a delay of −10 to −8 (τ = −10 to −8) and finishing at –2 to 0, where 0 is the point at which the animal left the startbox and entered the arena. For each 2s window (Trial × N × T_SB,τ_), a GNB decoder was run using a 10-fold cross validation strategy using a 70:30 train: test split. Correct trials were subsequently compared to incorrect trials using the temporal window of τ =−2 (the time during which the animal was most likely to be looking out into the arena) and a 1,000-fold cross-validation strategy.

Performance was evaluated by calculating the fraction of correctly predicted sandwells based on the actual label. Furthermore, the F1-score was calculated from the precision (the number of true positive results divided by the number of all positive results) and recall (the number of true positive results divided by the number of all samples that should have been identified as positive); see Eq. 4.

[5] $$\text{F}1=2\times \frac{\left(\mathrm{precision}\times \mathrm{recall}\right)}{\left(\mathrm{precision}+re\text{c}\mathrm{all}\right)}$$

Finally, for each training set, a comparable control was computed. This was achieved by training the GNB using X labeled with shuffled sandwell classes. Decoder performance was then tested on the same test sets as described above, generating a performance or F1-score expected at chance level (∼33%).

#### Cosine distance.

For each animal, all neurons that were active in the outbound trajectory of correct sample or choice trials were collated into the data structure (trial × N × T_outbound_ _correct_) and their neural event trains convolved with a Gaussian kernel (σ = 200 ms, window width of 4σ) to obtain a time series of instantaneous event rates. The cosine distance adjacency matrix $D_{C}\in T\times T$ was subsequently computed by treating each point in time as neural population vector (*x_i_*) with dimension N and calculating its cosine distance from all other time points within and across all trials (as shown in Eq. 5).

[6] $$D_{C}\left(x_{i},x_{i+n}\right)=1-\frac{x_{i}\cdot x_{i+n}}{\left|x_{i}\right|\left|x_{i+n}\right|}$$

#### MDS.

Dimensionality reduction or neural manifold learning takes a high dimension neural activity matrix, X∈N×T, and transforms it into the low dimension mapping Y∈k×T (where k ≪ N). Classical MDS is a linear dimensionality reduction algorithm based on based on the MATLAB cmdscale function applied to the previously computed cosine distance matrix $D_{C}\in T\times T$ to yield the lower dimension embedding Y∈k×T. This is computed through the minimization of a strain parameter that aims to preserve the relative cosine distances of *D_C_* in the manifold embedding Y. In parallel to this, an eigen-decomposition of the distance matrix *D_C_* is performed, with the resultant eigenvalues (or dimensions) sorted according to how much of the variance they explained with the top three dimensions selected for visualization.

#### Event rate.

For correct and incorrect sample and choice trials, neurons that were active in the 10-s period before the animal left the startbox up until 2 s into the arena were isolated and the event rate over time calculated. Event rate was calculated as the number of events per seconds.

#### Fraction of cell firing inside trajectory.

The arena was first binned into 4- × 4-cm spatial bins. A “trajectory mask” was then constructed using sample and choice trials, selecting bins that the animal traversed when leaving from the startbox to each of the three sandwells and back. The trajectory mask was then applied to all sessions and stages with any spatial activity outside the trajectory mask considered to be part of the “arena mask” (note, only bins that were visited were not included in the analysis). The fraction off cells firing inside the trajectory was subsequently calculated as the fraction of times a cell fired in the trajectory mask vs. the arena mask, normalized by the animal’s bin occupancy.

#### Angle of cell firing.

For each trial of sample and choice, we selected the cells active in the 10 s before leaving the startbox. We calculated the angle of cell firing θ as the average angle between the startbox door and the events recorded in the arena. Following convention in circular statistics, the origin was in the nominal east direction and positive angles were calculated anticlockwise so that a cell firing on the wall on the right of the door of the startbox has θ = 0°. The distribution was calculated for the cells active in the startbox in trials where the correct sandwell was 1, 2, or 3.

#### Cell trajectory classification.

For each trial of sample and choice, we selected the cells active in the 10 s before leaving the startbox. For each cell, we plotted the event positions of the neuron activity in the arena as recorded in the pretraining, sample, and choice phases. Each instance of prospective coding (cell, time) was classified as one of the sandwells if the event position was located either on the sandwell or in the trajectory between the startbox and the sandwell. In a minority of instances where pretraining events differed from the sample/choice (4.9%), mainly because of additional events outside the possible trajectories, only the sample and choice events were considered. Where the events mapped onto an area that did not correspond to any of the sandwells, the prospective coding event was classified as other (3.9%) and were not taken into account in the final calculations. The fraction of prospective coding for the correct sandwell was calculated as *n*_correct_/(*n*_correct_+*n*_incorrect_) where *n* is the number of prospective coding occurrences. Compound prospective coding events was considered if multiple cells represented the same sandwell (either as goal or trajectory) and were part of consecutive prospective coding events (≤100 ms), i.e., two prospective coding events were considered to be part of the same compound event if they were happening on the same time frame or the following one after binning at 100 ms. Clustering into compound prospective coding events was linearly additive; if coding events occurred at *t*, *t*+100ms, or *t*+200ms, a single compound prospective coding event of length = 3 was considered.

### Software.

Confocal and microscope images were open and processed with Fiji/ImageJ v2.1 (NIH); linear transformation of brightness and contrast was applied uniformly and equally to all compared images or channels. Calcium video processing was performed as described above using IDPS v1.6 and publically available or custom Python code. Statistical analysis was performed with GraphPad Prism v7.

### Note.

While this manuscript was in preparation, an independent report of calcium imaging in rats running along a linear track was published (27). This report showed that a high proportion of cells can be PCs in such an apparatus. Our study confirms this finding by using a rigorous mutual information criterion to identify PCs and goes on to deploy this imaging technology to gather insight into hippocampal function during decision-making.

Spatial recency within a well-learned context can be encoded and accessed remarkably fast. These observations complement those in another report, which was published while this manuscript was in preparation, to the effect that hippocampal representations can emerge after only a limited number of trials (48).

## Supplementary Material

Supplementary File

## Data Availability

All study data are included in the article and/or *SI Appendix*. The data are available at https://datashare.ed.ac.uk/handle/10283/4760. The software used for data analysis is available at https://github.com/rufusmitchellheggs/Neural-Predictive-Spatial-Coding (60).
