# Supplementary material for: Neuronal signature of spatial decision-making during navigation by freely moving rats by using calcium imaging
Source: Proc Natl Acad Sci U S A. 2022 Oct 24;119(44):e2212152119. doi: 10.1073/pnas.2212152119 (PMC9636941; doi:10.1073/pnas.2212152119)
Supplement: Supplementary File [file pnas.2212152119.sapp.pdf]

Supplementary Information and Figures

Neuronal signature of spatial decision-making during navigation by freely moving rats using calcium imaging

Francesco Gobbo[1,*], Rufus Mitchell-Heggs[1,2,*], Dorothy Tse[1,3], Meera Al-Omrani[4], Patrick Spooner[1,5], Simon R Schultz[2], Richard G.M. Morris[1,5]

[1]Centre for Discovery Brain Sciences, Edinburgh Neuroscience, The University of Edinburgh, EH8 9JZ Edinburgh (UK)

[2]Department of Bioengineering and Centre for Neurotechnology, Imperial College London, London, UK

[3]Department of Psychology, Edge Hill University, Ormskirk, UK

[4]MSc Program in Integrative Neuroscience, University of Edinburgh, EH8 9JZ Edinburgh (UK)

[5]Simons Initiative for the Developing Brain, University of Edinburgh

[*]co-first authors

Supplementary Discussion

Surgical considerations. While CA3-CA1 connections form the intrahippocampal Schaffer collaterals, extrahippocapal connections reach the hippocampus via three major routes: the fimbria/fornix, the angular bundle/perforant path and the temporoammonic alvear pathway. The fimbria/fornix contains the main bundle of afferent and efferent connections to and from subcortical regions. Most connections from the entorhinal cortex (EC) reach the hippocampus either via the performant path (pp) or the alvear path (ap) (1). The angular bundle is located between the EC and the presubiculum and parasubiculum. It is the main route taken by axons originating in the EC as they travel to the septotemporal levels of the other hippocampal fields, particularly the dentate gyrus, hippocampus and subiculum. These projections enter the hippocampal formation by perforate the subiculum (pp), proceeding intrahippocampally to innervate the dentate gyrus (DG) and CA3 and CA1 neurons (2). In CA1 pyramidal neurons, EC projections are disproportionally located in the stratum lacunosum moleculare adjacent to the DG (3). In our aspiration procedure, the Shaffer collateral, the fimbria and perforant path are unaffected, so that the main afferent paths to the CA1 region remain intact.

However, at more septal levels, the number of entorhinal fibers that take the alvear pathway is higher (4). In particular, in the septal portion of the hippocampal most of the EC fibers reach CA1 via the alveus by travelling parallel to the alveus then perforating the pyramidal cell layer (5). Because these fibers are medial to the location of our GRIN lens implant, the majority of the EC inputs to the hippocampus should be spared (see Figure SD1 below redrawn from Deller et al. 1996, showing EC anterograde tracts).

SD1

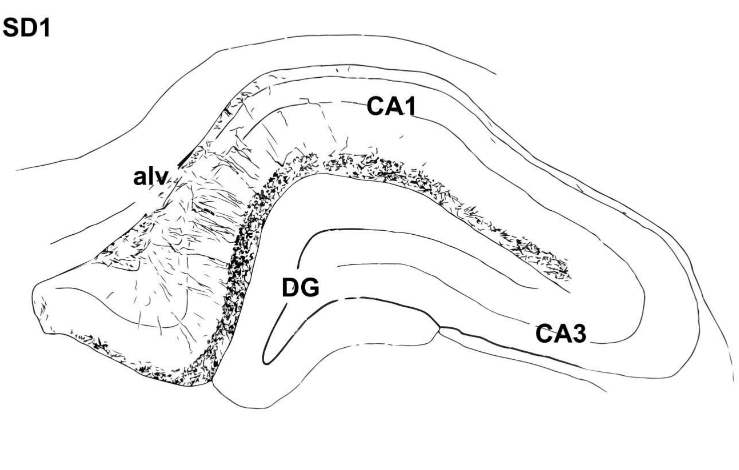

It has been reported that place cells activity and ripple generations are relatively robust to even large disruptions of EC inputs such as MEC lesions (6, 7), which would suggest that the majority of sensory inputs to the

hippocampus would be spared by the surgical procedure to image the rat CA1 and the placement of the small 0.9 mm GRIN lens. Nevertheless, in experiments where EC inputs are critical (8) this aspect should be taken into consideration. Indeed, the procedure described by (9) does report that cell identification is possible without aspirating the alvear fibers, albeit maybe at the expense of a lower effective resolution due to light scattering through myelinated fibers. Nonetheless, we and the authors of the other study (9) will have concerns about damage to stratum oriens but our histology shows minimal damage. A side-by-side comparison of the two technical approaches (aspirating vs keeping alvear fibers) would probably be useful.

The aspiration of callosal fibers is, however, necessary for a successful imaging, as also in (9). The corpus callosum contains commissural fibers connecting, among other regions, the hippocampal formation between the two hemispheres. The position of the GRIN lens implant is expected to spare the dorsal hippocampal commissure connecting the dorsal hippocampi, but it cannot be excluded that the implant can affect interhippocampal communication. Although poorly studied with respect to this aspect, the rat hippocampus seem to have little lateralization; moreover, interhemispheric communication does not seem to severely impact the performance in mnemonic tasks (10, 11), but see (12).

Behaviour setup considerations. The everyday arena allows to model episodic-like memories in rodents (13, 14). A key facet of the task is that the position of the reward changes from day to day in an unpredictable way. However, the global spatial context remains unchanged. Thus, on any day, there is nothing to learn about the spatial context and we therefore had every reason to expect place cell fields to remain stable. The animal does, however, have to update its representation of the most recent spatial location and not use a consolidated spatial memory.

In our experiment, we used a modified version of the everyday arena task to accommodate the requirements of calcium imaging, mainly to maintain 3 possible sandwells. Although the sandwells are not at equal distance from the animals, this was deliberate to avoid symmetry and we see no reason why this might bias the animal's preference or its patterns of cell firing. It is now common practice in the everyday arena task to use multiple sandwells configurations in the same experiments (14).

In replay experiments using electrophysiology, a plethora of maze configurations have been used. Linear tracks and variants thereof (such as Z-tracks) are by far the most commonly used experimental setups (15, 16). Two-

choice or multiple-choice W mazes have also been employed, where the distance of the possible end goals is typically equivalent from the animal's starting point (17–19). These configurations can be easily modelled and linearized in the analysis (19) although at the expense of limiting the choices of the animals in solving the task. As discussed in the main text, this may also bias how much animals focus on the different locations, which can be reflected in the neural reactivation. Other experiments have privileged a less structured behavioural setup, which allows the animals greater flexibility during the decision making, and allows for a more naturalistic behaviour. In such cases, two-dimensional environments are typically chosen, with either a grid-like or a random (or quasi-random) distribution of the goals (20–23). These reflect the random positioning of goals with respect to the animals' starting points, as is the case in the most widely used behavioural setup to study navigation and spatial memory such as the Barnes maze, the watermaze and the Cheeseboard maze (20, 24). In our experiment, we chose the latter approach, and indeed our sandwell configuration is very close to the one used in (20). Future work will address if, and to what extent, the configuration of the arena or other experimental features affect the representation of space and the prospective coding during decision making.

Supplementary References

1. M. P. Witter, F. G. Wouterlood, P. A. Naber, T. Van Haeften, Anatomical Organization of the Parahippocampal-Hippocampal Network. *Annals of the New York Academy of Sciences* **911**, 1–24 (2006).

2. D. G. Amaral, H. Kondo, P. Lavenex, An analysis of entorhinal cortex projections to the dentate gyrus, hippocampus, and subiculum of the neonatal macaque monkey: Entorhinal projections to the hippocampus in neonatal monkeys. *J. Comp. Neurol.* **522**, 1485–1505 (2014).

3. R. Kajiwara, *et al.*, Convergence of entorhinal and CA3 inputs onto pyramidal neurons and interneurons in hippocampal area CA1 - An anatomical study in the rat. *Hippocampus* **18**, 266–280 (2008).

4. T. Deller, G. Adelmann, R. Nitsch, M. Frotscher, The alvear pathway of the rat hippocampus. *Cell and Tissue Research* **286**, 293–303 (1996).

5. P. Anderson, R. G. M. Morris, D. Amaral, T. Bliss, J. O'Keefe, *The Hippocampus Book* (2007) https:/doi.org/10.1007/PL00012130.

6. J. B. Hales, *et al.*, Medial Entorhinal Cortex Lesions Only Partially Disrupt Hippocampal Place Cells and Hippocampus-Dependent Place Memory. *Cell Reports* **9**, 893–901 (2014).

7. A. Chenani, *et al.*, Hippocampal CA1 replay becomes less prominent but more rigid without inputs from medial entorhinal cortex. *Nature Communications* **10** (2019).

8. T. J. Bell, *et al.*, Intron retention facilitates splice variant diversity in calcium-activated big potassium channel populations. *Proceedings of the National Academy of Sciences of the United States of America* **107**, 21152–21157 (2010).

9. H. S. Wirtshafter, J. F. Disterhoft, *In Vivo* Multi-Day Calcium Imaging of CA1 Hippocampus in Freely Moving Rats Reveals a High Preponderance of Place Cells with Consistent Place Fields. *J. Neurosci.*, JN-RM-1750-21 (2022).

10. J. T. Jordan, The rodent hippocampus as a bilateral structure: A review of hemispheric lateralization. *Hippocampus* **30**, 278–292 (2020).

11. Y. Sakaguchi, Y. Sakurai, Disconnection between Rat's Left and Right Hemisphere Impairs Short-Term Memory but Not Long-Term Memory. *Symmetry* **13**, 1872 (2021).

12. S. Klur, *et al.*, Hippocampal-dependent spatial memory functions might be lateralized in rats: An approach combining gene expression profiling and reversible inactivation. *Hippocampus* **19**, 800–816 (2009).

13. T. Bast, B. M. Da Silva, R. G. M. Morris, Distinct contributions of hippocampal NMDA and AMPA receptors to encoding and retrieval of one-trial place memory. *Journal of Neuroscience* **25**, 5845–5856 (2005).

14. M. Nonaka, *et al.*, Everyday memory: towards a translationally effective method of modelling the encoding, forgetting and enhancement of memory. *European Journal of Neuroscience* **46**, 1937–1953 (2017).

15. C.-T. Wu, D. Haggerty, C. Kemere, D. Ji, Hippocampal awake replay in fear memory retrieval. *Nat Neurosci* **20**, 571–580 (2017).

16. H. F. Ólafsdóttir, F. Carpenter, C. Barry, Task Demands Predict a Dynamic Switch in the Content of Awake Hippocampal Replay. *Neuron* **96**, 925-935.e6 (2017).

17. S. P. Jadhav, C. Kemere, P. W. German, L. M. Frank, Awake hippocampal sharp-wave ripples support spatial memory. *Science* **336**, 1454–1458 (2012).

18. M. P. Karlsson, L. M. Frank, Awake replay of remote experiences in the hippocampus. *Nature Neuroscience* **12**, 913–918 (2009).

19. A. K. Gillespie, *et al.*, Hippocampal replay reflects specific past experiences rather than a plan for subsequent choice. *Neuron* **109**, 3149-3163.e6 (2021).

20. D. Dupret, J. O'Neill, B. Pleydell-Bouverie, J. Csicsvari, The reorganization and reactivation of hippocampal maps predict spatial memory performance. *Nature Neuroscience* **13**, 995–1002 (2010).

21. B. E. Pfeiffer, D. J. Foster, Hippocampal place-cell sequences depict future paths to remembered goals. *Nature* **497**, 74–79 (2013).

22. A. Berners-Lee, *et al.*, Hippocampal replays appear after a single experience and incorporate greater detail with more experience. *Neuron*, 1–14 (2022).

23. J. Widloski, D. J. Foster, Flexible rerouting of hippocampal replay sequences around changing barriers in the absence of global place field remapping. *Neuron* **110**, 1547–1558 (2022).

24. R. G. M. Morris, Spatial localization does not require the presence of local cues. *Learning and Motivation* **12**, 239–260 (1981).

Table S1 *Statistical information*

Figure 1l	N=8 rats	Repeated measures one-way ANOVA,<br>Session effect P=0.0339, DF=21, F(4485,31.4)=2.881<br>Individual effect P=0.4708, DF=7, F(7,147)=0.949<br>Geisser-Greenhouses's epsilon = 0.2136, R^2=0.2916<br><br>Post-test for trend,<br>Slope 0.0155, P<0.0001, F(1,147)=41.95<br><br>Sessions 18-21<br>average 0.906, sem 0.035<br>D'Agostino Pearson normality test K2=3.603, P=0.165<br>Student's t-test to 0.5: t = 11.61, df=7, P<0.0001
Figure 2e	N=5 rats	Sandwell 1 Data vs Shuffle<br>Student's t-test to 0.5: t =2.96, df=8, P=0.0181<br><br>Sandwell 2 Data vs Shuffle<br>Student's t-test to 0.5: t =4.56, df=8, P=0.0019<br><br>Sandwell 3 Data vs Shuffle<br>Student's t-test to 0.5: t =3.01, df=8, P=0.0167<br><br>All cells Data vs Shuffle<br>One-way ANOVA<br>Between effect F(1,8)= 45.4678, df=1, P<0.0001<br>Student's t-test to 0.5: t =6.77, df=8, P<0.0001<br><br>Place cells Data vs Shuffle<br>Between effect F(1,8)= 16.7011, df=1, P= 0.0035<br>Student's t-test to 0.5: t =4.12, df=8, P=0.0034<br><br>Spatially selective cells Data vs Shuffle<br>Between effect F(1,8)= 3.7510, df=1, P= 0.3192<br>Student's t-test to 0.5: t =1.98, df=8, P=0.0834<br><br>All cells Data vs Place cells Data<br>Between effect F(1,8)= 0.6518, df=1, P= 0.44281

		Student’s t-test to 0.5: t =0.83, df=8, P=0.4331<br><br>All cells Data vs Spatially selective<br>Between effect F(1,8)= 17.8554, df=1, P= 0.0029<br>Student’s t-test to 0.5: t =4.517, df=8, P=0.0020<br><br>Place cells Data vs Spatially selective cells Data<br>Between effect F(1,8)= 7.7732, df=1, P=0.0236<br>Student’s t-test to 0.5: t =2.92, df=8, P=0.0193
Figure 3c	N=5 animals	One-way ANOVA<br>F(3,14)=43.84, df=3, P<0.0001<br>Bartlett’s test 6.512 P=0.0892<br>Brown-Forsythe test F(3,14)=0.7436 P=0.5437<br><br>Correct Trials vs Control<br>D=-0.6258 CI=(-0.829,-0.4226) P<0.0001<br><br>Correct Trials vs Incorrect Trials<br>D=0.7378 CI=(0.5223,0.9533) P<0.0001<br><br>Correct Trials vs Incorrect Trials, Actual sandwell<br>D=0.4085 CI=(0.4085,0.8395) P=<0.0001<br><br>Incorrect Trials vs Control<br>D=-0.112 CI=(-0.3275,0.1035) P=0.4577<br><br>Incorrect Trials, Actual Sandwell vs Control<br>D=0.0018 CI=(-0.3275,0.1035) P=0.9999<br><br>Incorrect Trials, Actual Sandwell vs Incorrect Trials<br>D=0.2404 CI=(-0.1138,0.07816) P=0.4878
Figure 3f	N=5 animals	Kolmogorov-Smirnov test<br>Sandwell 1 vs Sandwell 2<br>D=0.3989 P=3.33E-16

		Sandwell 1 vs Sandwell 3<br>D=0.1985 P=1.16E-12<br><br>Sandwell 2 vs Sandwell 3<br>D=0.2134 P=7.44E-24
Figure 3g	N=5 animals	Ratio paired t-test, two tailed<br>t=5.406, df=4 P=0.0057
Figure 3h	N=4 animals (one animal did not have incorrect trials)	Ratio paired t-test, two tailed<br>t=1.239, df=3 P=0.3033
Figure S4b	N=5 animals	Mean±sem : 0.9436±0.0065<br>z-test to 0:<br>t=145.5 df=4, P<0.0001
Figure S4c	N=5 animals	NeuN<br>Mean±sem : 1.000±0.000<br><br>GAD67<br>Mean±sem : 0.0026±0.0011<br>z-test to 0:<br>t=2.428 df=4, P=0.0721
Figure S5 b	N=8 animals	Repeated measures one-way ANOVA,<br>Session effect P=0.6457, DF=21, F(5569,38.98)=0.694<br>Individual effect P=0.6488, DF=7, F(7,147)=0.7276<br>Geisser-Greenhouses's epsilon = 0.2652, R^2=0.0902<br><br>Post-test for trend,<br>Slope 0.01133, P=0.0037, F(1,147)=8.693<br><br>Sessions 18-21<br>average 0.844, sem 0.066<br>D'Agostino Pearson normality test K2=2.565, P=0.277

		Student’s t-test to 0.5: t = 5.227, df=7, P=0.0012
Figure S5 c	N=8<br>animals	Repeated measures two-way ANOVA,<br>Probe trial number (P1-P2) effect: P=0.8037, DF=1, F(1,14)=0.06416<br>Sandwell effect (correct-incorrect): P=0.0005, DF=1, F(1,14)=20.37<br>Interaction effect: P=0.4599, DF=1, F(1,14)=0.8037<br>Individual effect: P=0.9183, DF=14, F(14,14)=0.4643
Figure S13b	N=5<br>animals	One-way ANOVA<br>F(3,16)=16.44, df=3, P<0.0001<br>Bartlett’s test 9.614 P=0.022<br>Brown-Forsythe test F(3,16)=0.6556 P=0.5911<br><br>Correct Trials vs Control<br>D=-0.5705 CI=(-0.8352,-0.3059) P=<0.0001<br><br>Correct Trials vs Incorrect Trials<br>D=0.5041 CI=(0.2395,0.7688) P=0.0003<br><br>Correct Trials vs Incorrect Trials, Actual sandwell<br>D=0.5059 CI=(0.2412,0.7705) P=0.0003
Figure S15a	N=5<br>animals	Ratio Paired t-test<br>t=5.976 df=4 P=0.0039
Figure S15d	N=5<br>animals	Ratio Paired t-test<br>t=3.459 df=4 P=0.0258
Figure S19a	N=5<br>animals	Kolmogorov-Smirnov test<br>Sandwell 1 vs Sandwell 2<br>D=0.1397 P=7.898E-10<br><br>Sandwell 1 vs Sandwell 3<br>D=0.1486 P=1.22E-15<br><br>Sandwell 2 vs Sandwell 3<br>D=0.1864 P=1.21E-18
Figure S20a	N=5<br>animals	Correct Choice trials length<br>Rewarded vs non-rewarded

		Ratio Paired Student's t-test, t=4.372 df=4 p=0.0119
Figure S20b	N=5 animals	Direct Sample trials length<br>Rewarded vs non-rewarded<br>Ratio Paired Student's t-test, t=4.276 df=4 p=0.0129
Figure S20c	N=5 animals	Repeated Measures one-way ANOVA<br>SAMPLE:<br>Geisser-Greenhouse's epsilon=0.4524<br>Trial effect df=5 F(2.262 ,9.049) P=0.1140<br>Test for trend slope= 0.02717 P=0.0037 F(1,20)=10.77<br><br>CHOICE:<br>Geisser-Greenhouse's epsilon=0.4486<br>Trial effect df=5 F(2.243,8.973)=0.3705 P=0.7228<br>Test for trend slope =0.0142 P = 0.2742 F(1,20)=1.264

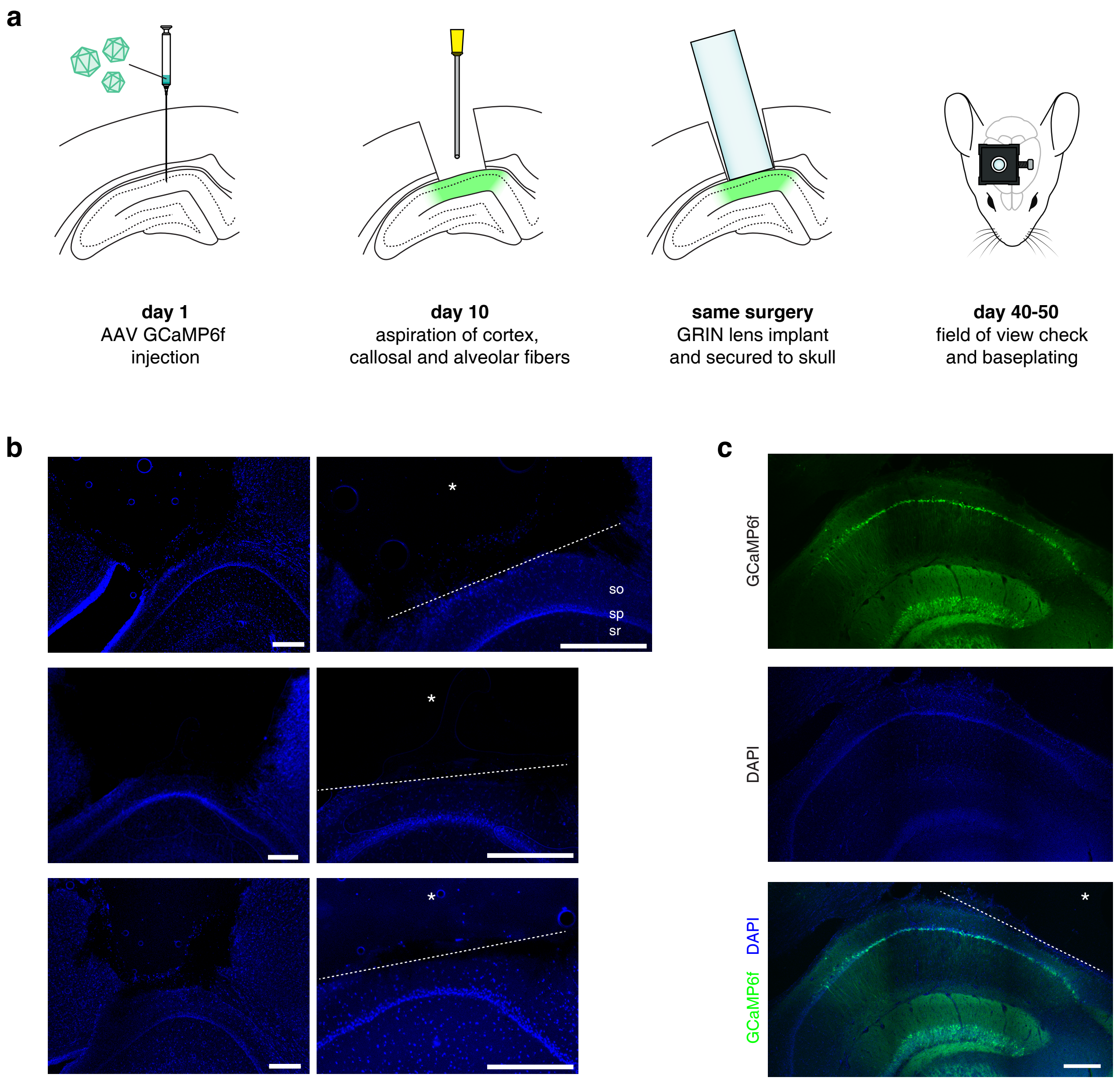

Figure S1 *Surgical procedure to image rat CA1*. (**a**) Schematic of the experimental protocol to express GCaMP6f and implant the GRIN lens on rat CA1. (**b**) Representative animals showing the aspiration and removal of cortical tissue and axonal fibers on top of CA1 after the successful implant of the GRIN lens (DAPI staining). The position of the former GRIN lens is marked with an asterisk, and the reconstructed inner surface of the GRIN lens is displayed a dashed white line. Panels on the right are higher magnification images of panels on the left. Scale bar 250µm. (**c**) Representative animal showing GCaMP6f endogenous fluorescence (green) and DAPI staining (blue) after aspiration and implant of the GRIN lens. Scale bar 250µm.

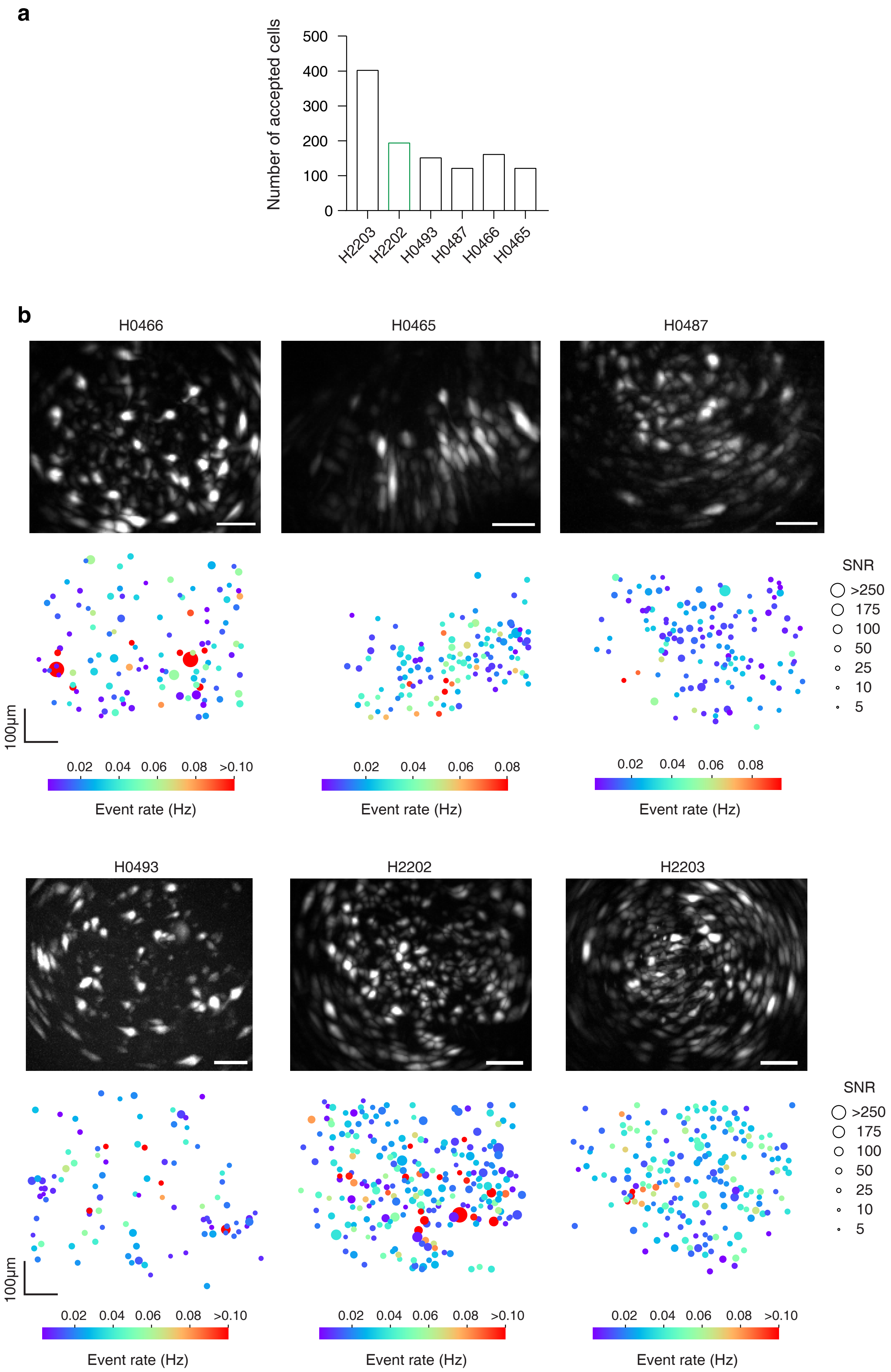

a
Number of accepted cells
500
400
300
200
100
0
H2203
H2202
H0493
H0487
H0466
H0465
b
H0466
H0465
H0487
SNR
>250
175
100
50
25
10
5
100µm
0.02
0.04
0.06
0.08
>0.10
Event rate (Hz)
H0493
H2202
H2203

Figure S2 *Calcium imaging in rat CA1*. (**a**) Number of cells detected for the 6 animals included in this study. The animal in green (H2202) is the animal displayed in Figure 1d-f. (**b**) Maps of accepted cells to display their average event rate and their signal-to-noise ratio. Maps correspond to S18 and are essential equivalent for Sessions 19-21.

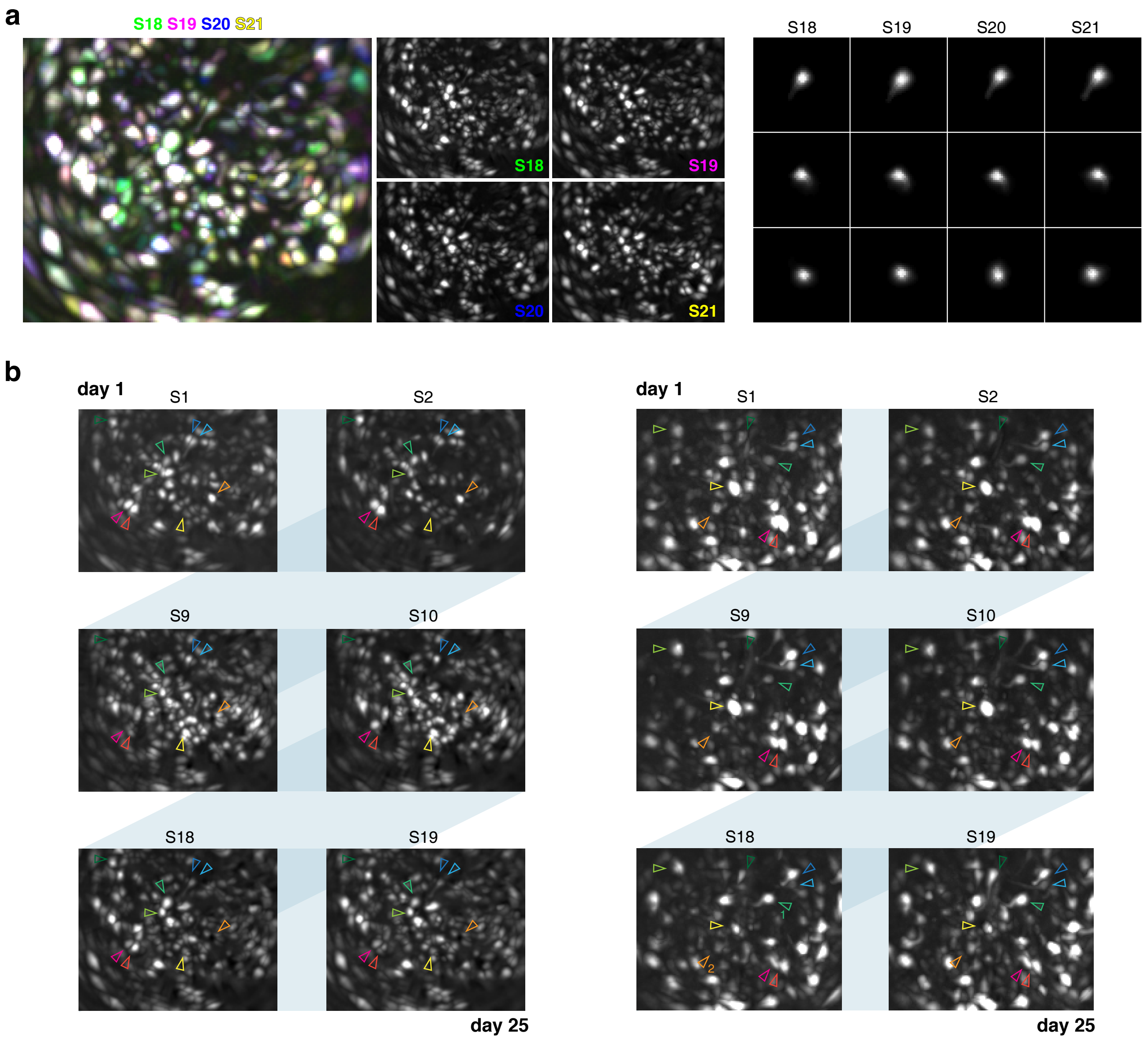

Figure S3 *Longitudinal registration*. (**a**) Projection maps for sessions 18-21 for the animal displayed in Figure 1d-f. (**b**) Longitudinal registration for a month of recording (sessions 1-2, 9-10 and 18-19) for two representative animals. Projected ΔF/F maps are displayed. Arrowheads of different colors highlight representative cells that can be followed across all sessions.

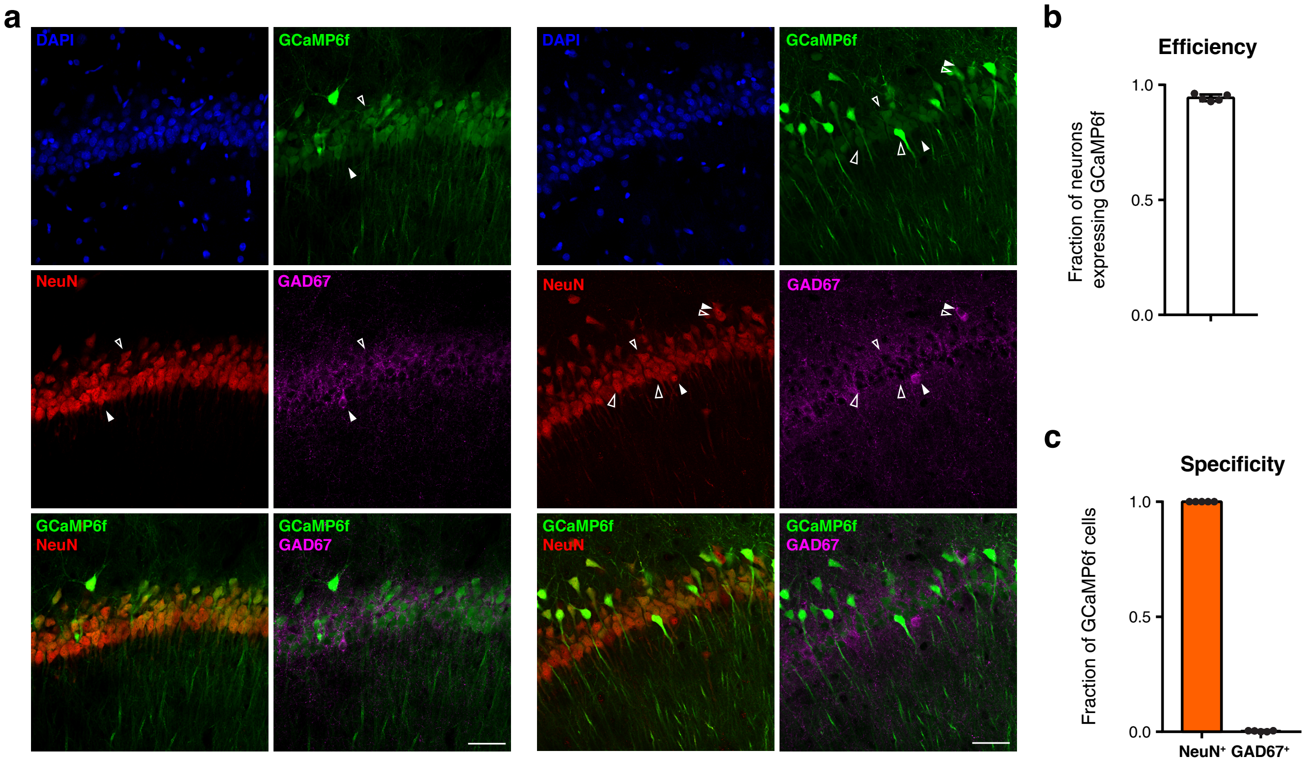

Figure S4 *Identification of recorded cell types*. (**a**) Two representative animals showing endogenous GCaMP6f signal (green) compared to pan-neuronal marker NeuN (red) and inhibitory neuron marker GAD67 (magenta), and counterstained for DAPI (blue). Scale bar 50μm. (**b**) Quantification of GCaMP6f efficiency in the central dorsal CA1 as a fraction of $NeuN^+$ neurons. (**c**) Quantification of the $NeuN^+$ and $GAD67^+$ GCaMP6f-expressing cells.

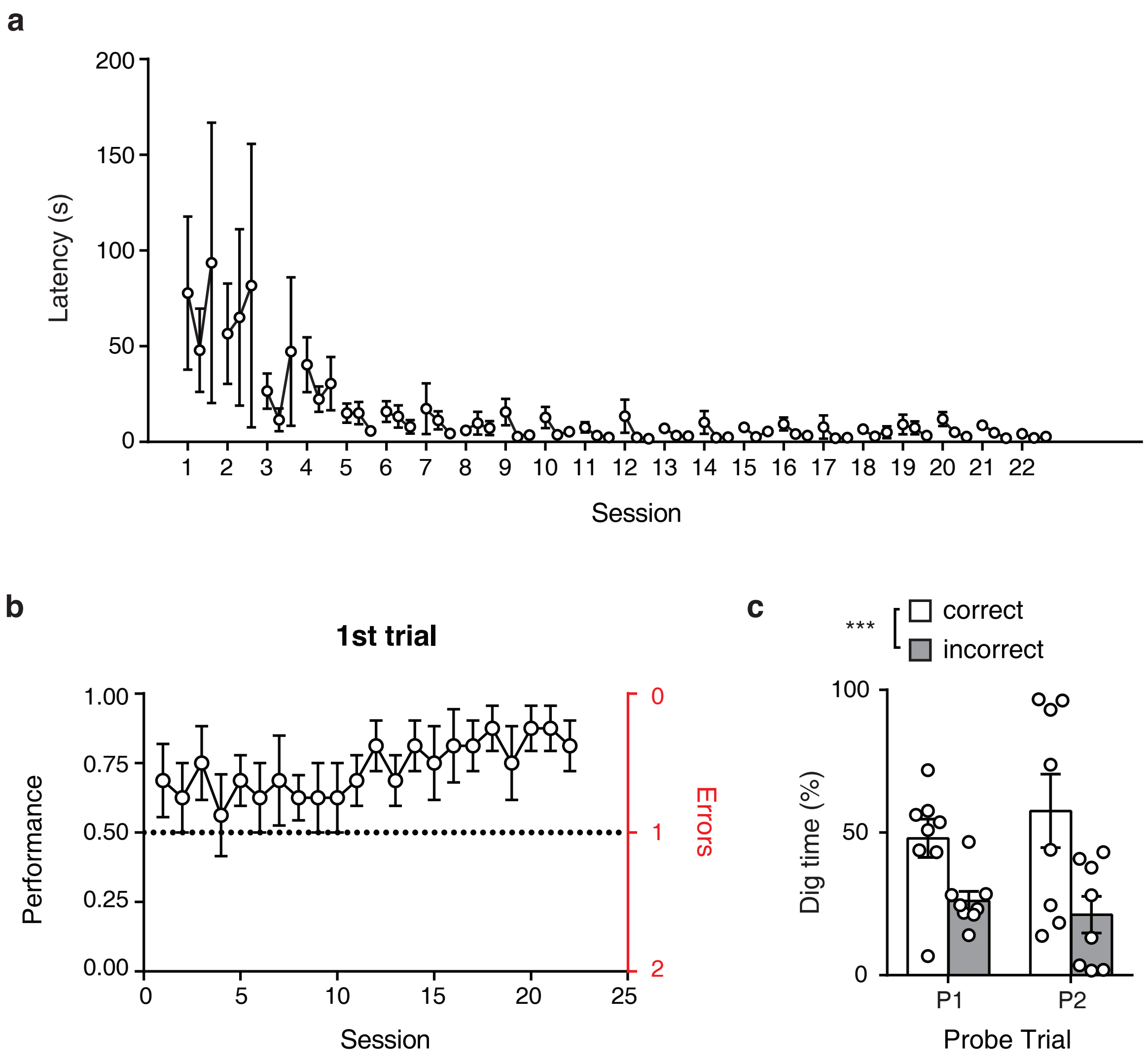

Figure S5 *Behaviour*. (**a**) Latency from the moment of leaving the startbox to reaching the correct Sandwell for the first 3 trials of the Choice phase in each session. Mean±sem. (**b**) Performance calculated for the first trial of each session in the Choice phase. (c) Percent digging time at the correct Sandwell during probe trials. ***P<0.001 two-way ANOVA, correct vs incorrect effect.

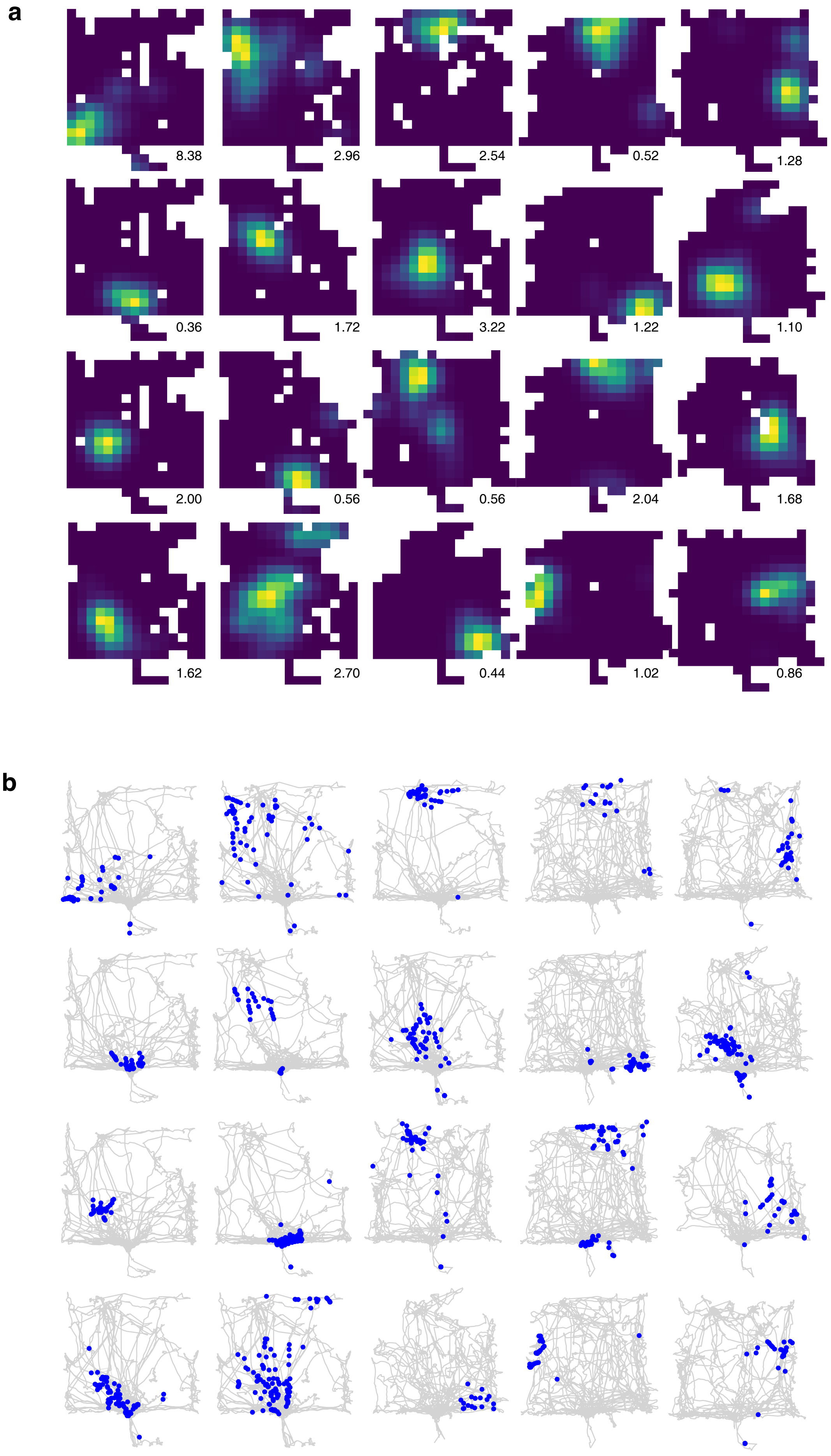
a
8.38
2.96
2.54
0.52
1.28
0.36
1.72
3.22
1.22
1.10
2.00
0.56
0.56
2.04
1.68
1.62
2.70
0.44
1.02
0.86
b

c

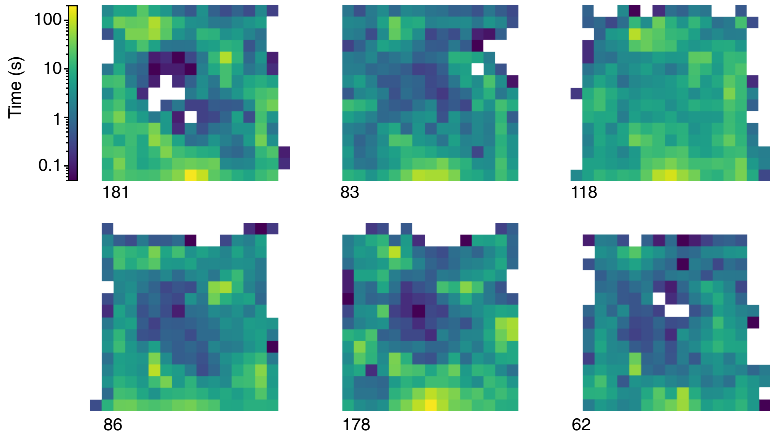

Figure S6 *Place cells and spatial occupancy across sessions*. (**a**) Representative place maps detected from different animals and different sessions. Number below each plot represents the peak value in events per second. (**b**) Corresponding event positions (blue dots) for the cells represented in (a). The animal trajectory is represented in gray. (**c**) Occupancy maps for 6 different animals showing the cumulative duration spent in each of the arena's spatial bins across all four 'Exploration' sessions. Number below each plot represents the peak bin duration.

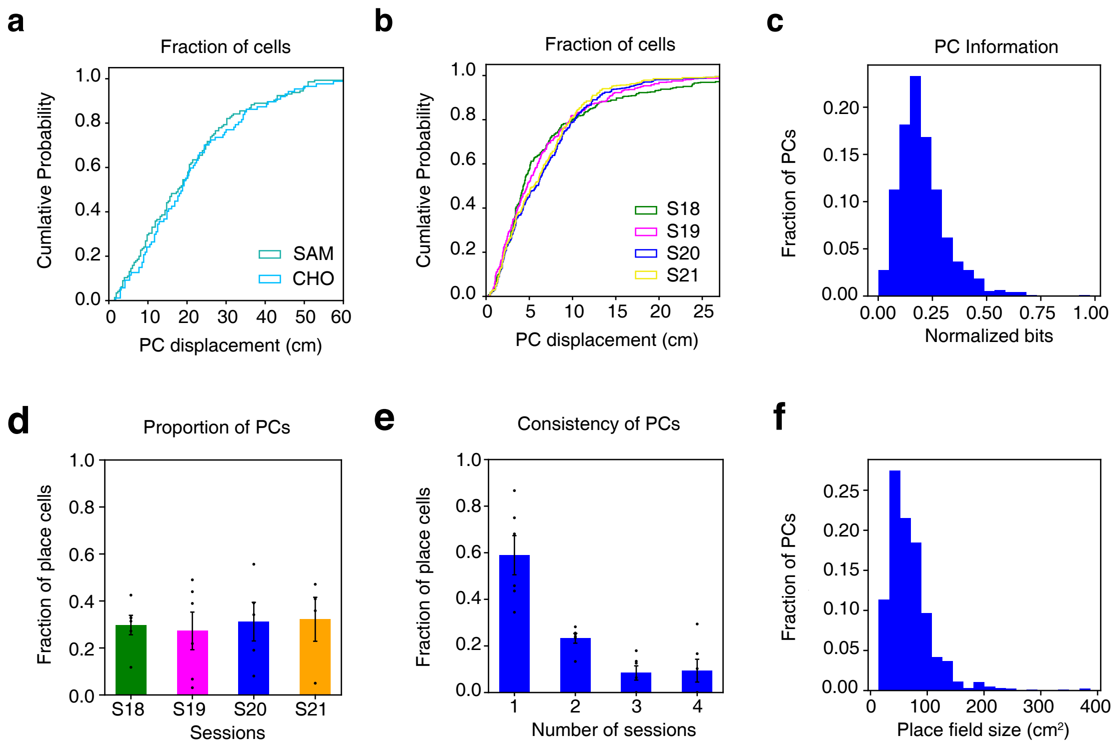

Figure S7 *Place cells properties*. (**a**) Cumulative distribution of place cell displacement in Sample and Choice phases compared to Exploration. (**b**) Cumulative distribution of place cell displacement compared to the first session where they have been detected. (**c**) Information per event of place cells activity. (**d**) Fraction of neurons classified as place cells in each session. (**e**) Number of cells classified as place cells in only session, in 2 sessions, in 3 or 4 sessions). (**f**) Distribution of place field sizes.

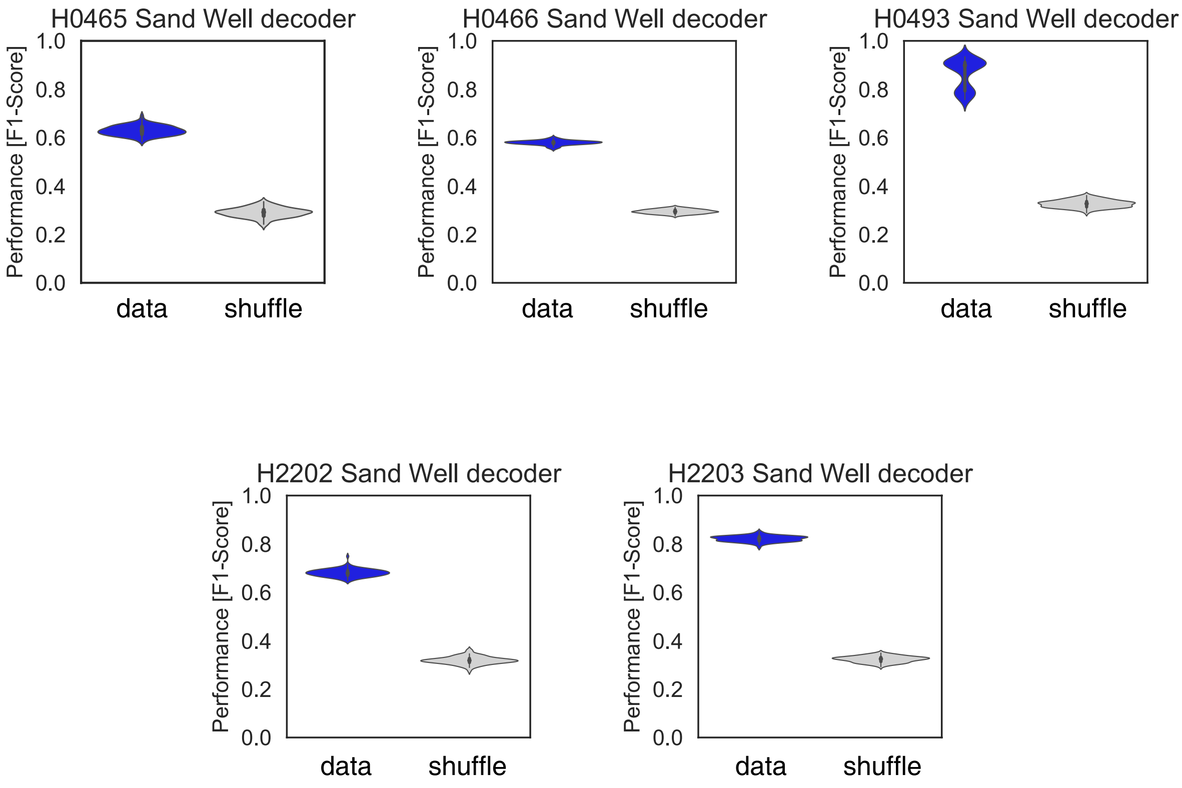

Figure S8 *Decoder of animal position*. Decoding performance of individual animals as displayed in Figure 2e. Data are in blue, and shuffled data in gray.

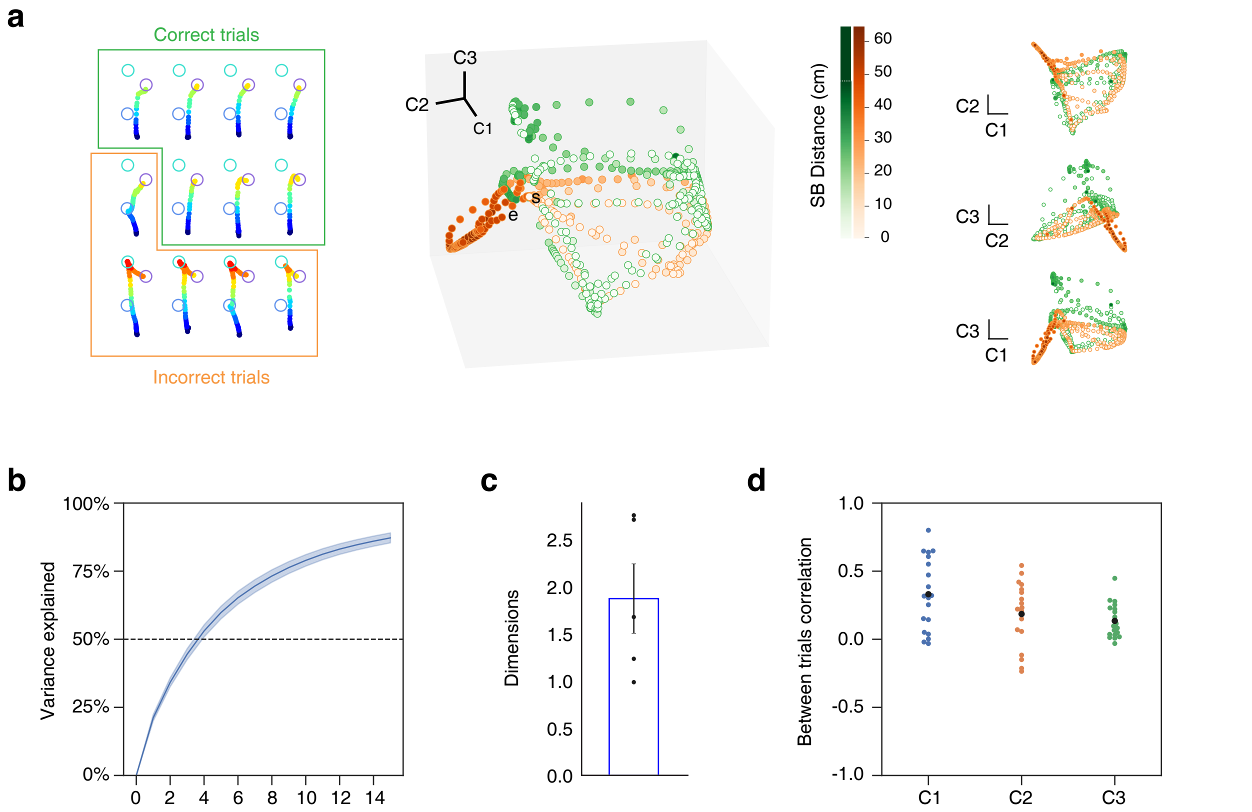

Figure S9 *Manifold*. (**a**) Correct and incorrect trials can be distinguished in the manifold space. On the left, behavioural trajectories for correct and incorrect trials. Middle and right, manifold representation in the 3 main Components of the corresponding neural activity. 's' and 'e' denote the start and the end of each trial (startbox to Sandwell). Correct trials are in shades of green, while incorrect one are in shades of orange as indicated. (**b**) Cumulative variance explained as a function of the number of components. (**c**) Average number of dimensions to explain 25% of the variance. (**d**) Correlation between trials for the first three manifold components.

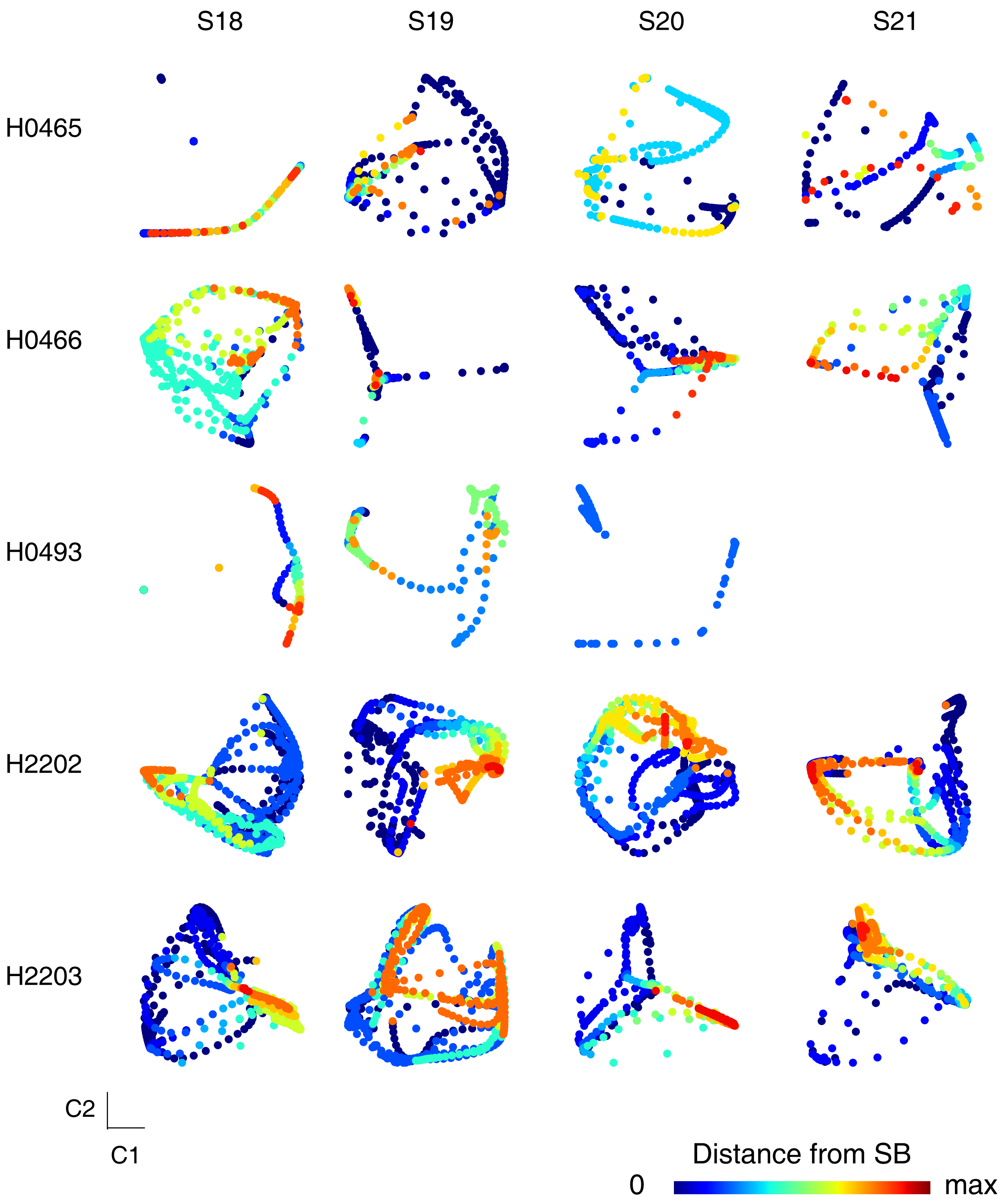

Figure S10 *Manifold*. (**a**) Manifold representation of the outbound parts of the trials for each session and animal (startbox/blue to Sandwell/red).

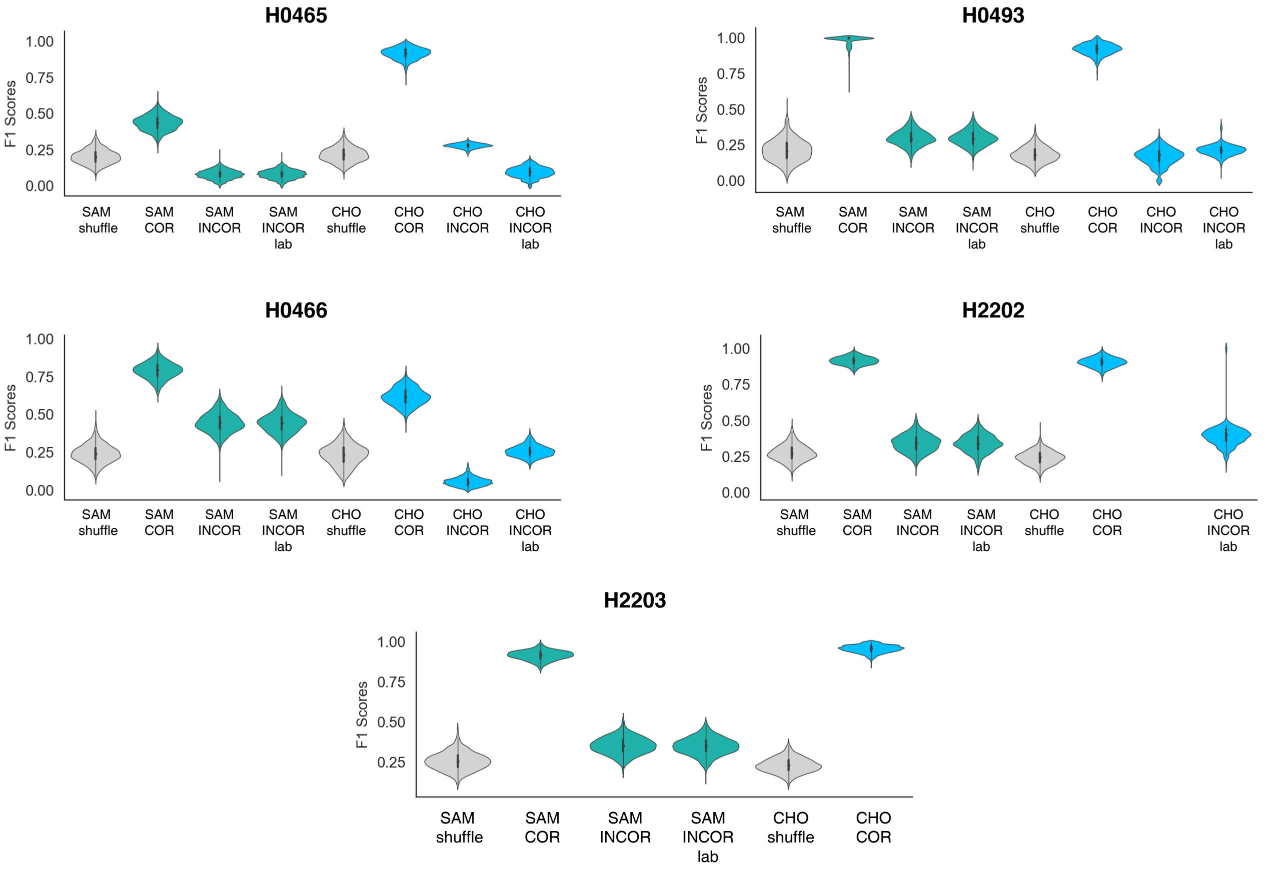

Figure S11 *Decoding destination from startbox activity*. Decoding performance for the individual animals shown in Figure 3c for choice (SAM, blue) and sample trials (CHO, green). As in the main figure, each dataset shows the performance for shuffled data, correct trials, incorrect trials, and incorrect trials relabelled to match the actual first visited Sandwell.

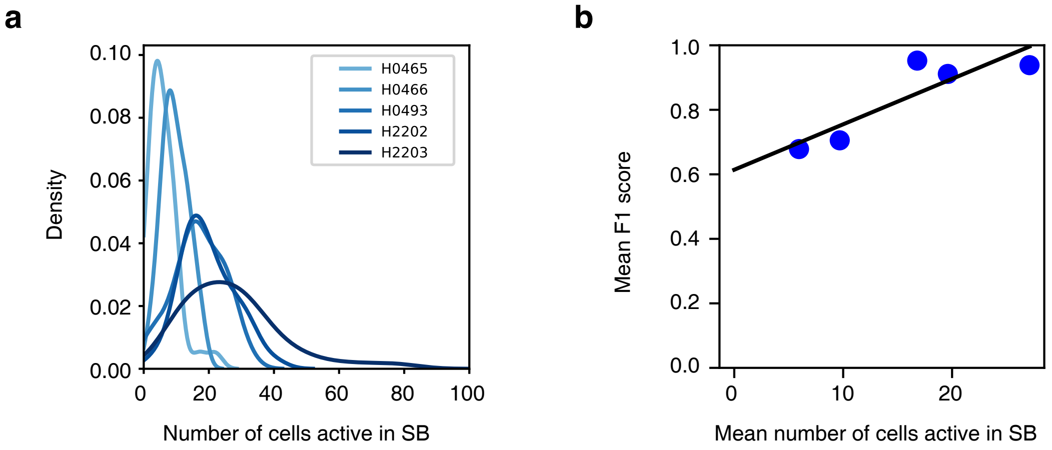

Figure S12 *Cell activity in the startbox*. (**a**) Distribution of number of cells active per trial in the startbox in the 10s before leaving for each animal. (**b**) Decoding performance plotted against the average number of active cells. Linear regression is plotted.

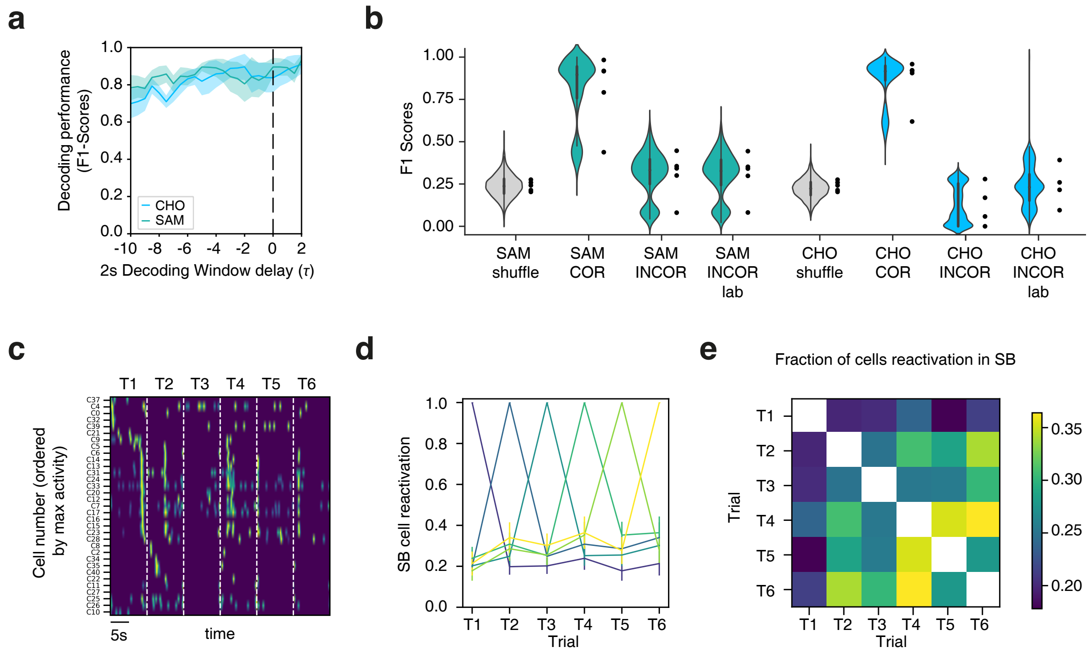

Figure S13 *Decoding performance*. (**a**) Decoding performance as in Figure 3b for sample (blue) and choice (green) trials. (**b**) As in Figure 3c including the average performance for Sample trials. See also Figure S11. ***P<0.0001 one-way ANOVA comparison of means. (**c**) Example of variable repetition of startbox activity in the 10s before each trial for one session. Cells are ordered by maximum activity. (**d,e**) Average number of common cells between trials in the same session.

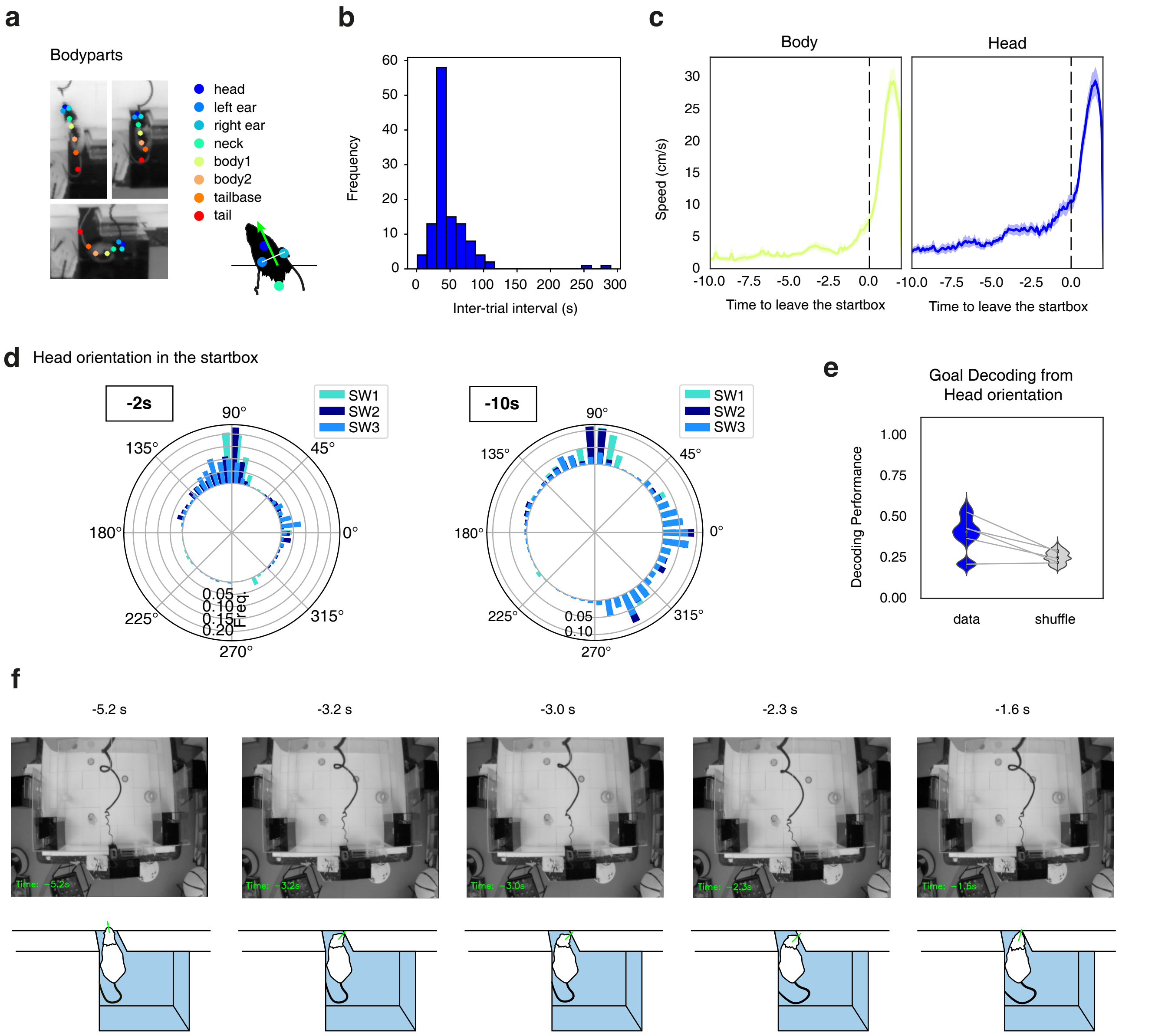

Figure S14 *Startbox behaviour.* (**a**) Detection of multiple body parts for the determination of the animal's head orientation in the startbox. Schematic of the head orientation calculation (green - vector). An animal facing East will have head orientation = 0° by convention. (**b**) Distribution of inter-trial interval length, the time elapsed between the end of one trial with the return to the startbox, and the beginning of the subsequent one. (**c**) Animal speed in the - 10 seconds to +2 seconds from leaving the startbox. Plotted is the speed for the body (left) and the head (right). The higher speed for the head likely reflects the animals turning their head while staying still. Plots are mean±sem. (**d**) Distribution of head orientations in the last 2 seconds before leaving the startbox (left) or the last 10s before leaving (right). Histograms are divided by trial goal based on the rewarded sandwell. (e) Decoding performance of a decoder trained to predict the goal sandwell based on the head direction of the animal in the startbox. (f) Video frames and head position reconstruction of the frames displayed in Figure 3d. The light blue area is the startbox area, and the head orientation is displayed as a green vector.

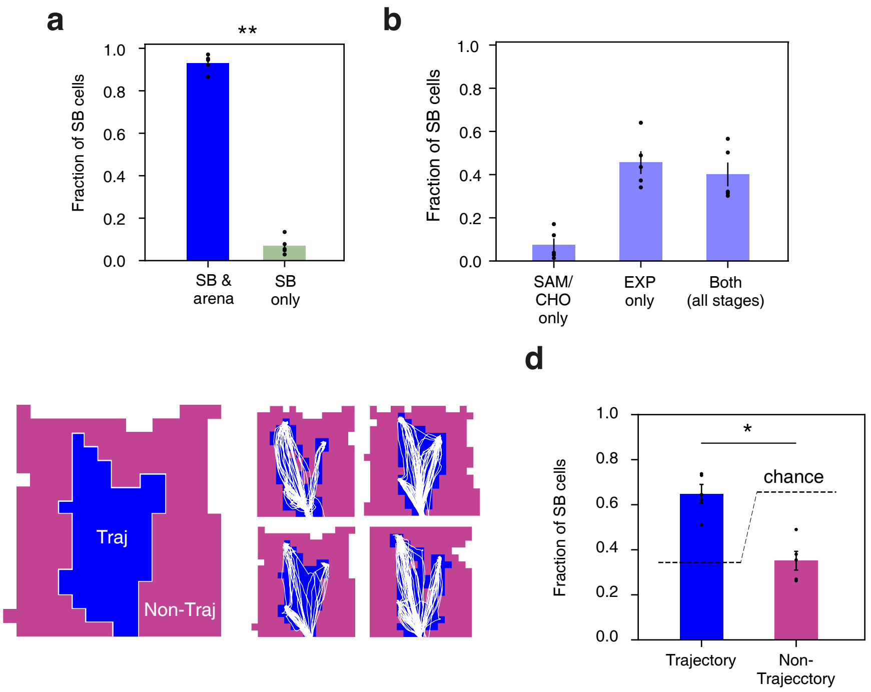

Figure S15 *Content of prospective coding*. (**a**) Fraction of cells active in the startbox that are also active in the arena or only in the startbox. **P<0.01 paired Student's t-test. (**b**) Breakdown of cells active in the startbox and in the arena in panel (a). Cells active in sample and choice but not exploration; cells active in exploration only; cells active in sample, choice, and exploration. (**c**) Representative area covered during sample and choice trials. The area covered during these phases was labelled as 'Trajectory' and the rest as 'Non-Trajectory' (left). An trajectory mask was calculated for each animal using SAM & CHO trials to and from the sandwells (right) (**d**) Position of events for cells active in the startbox during the 10s before leaving it. P<0.05 paired Student's t-test. Chance level is calculated for the random distribution taking into account the place occupancy.

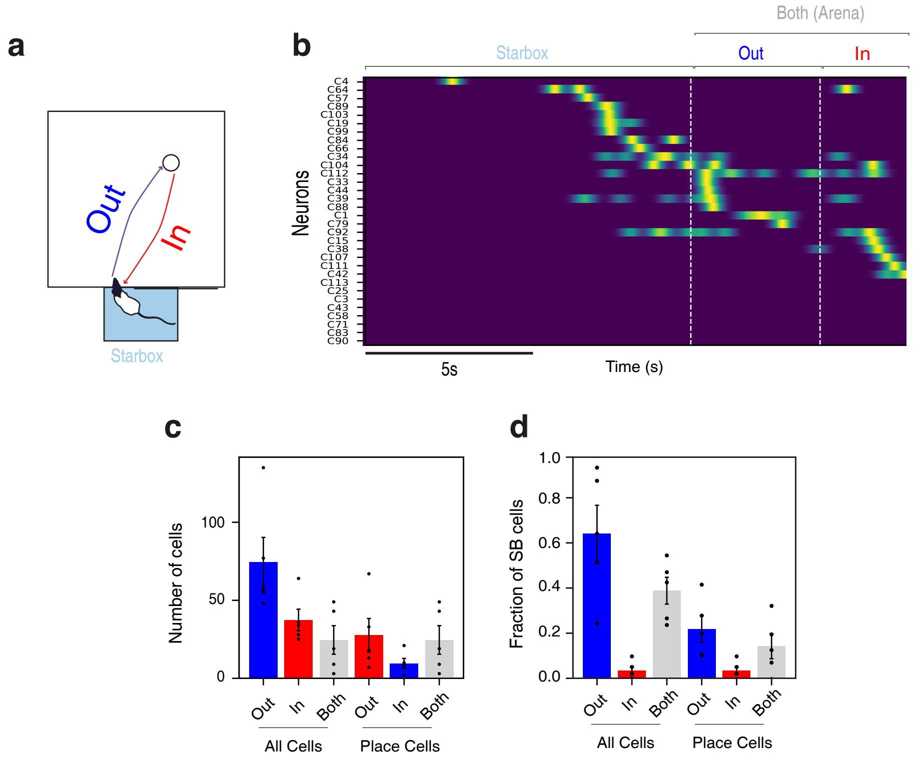

Figure S16 *Startbox cell reactivation in inbound vs outbound paths.* (**a**) Schematic of animal trial, showing "outbound" path to the sand well and "inbound" path back to the startbox. (**b**) Representative trial neural activity ordered by maximal activity, made up of 10s in startbox before the animal enters the arena, the outbound path and the inbound path. (**c**) Number of cells with events in the outbound path only, inbound path only and in both paths for "All Cells" and out of "Place Cells". (**d**) Fraction of cells with events in the startbox that also have events only in the outbound vs only in the inbound vs in both paths out of "All Cells" and out of "Place Cells".

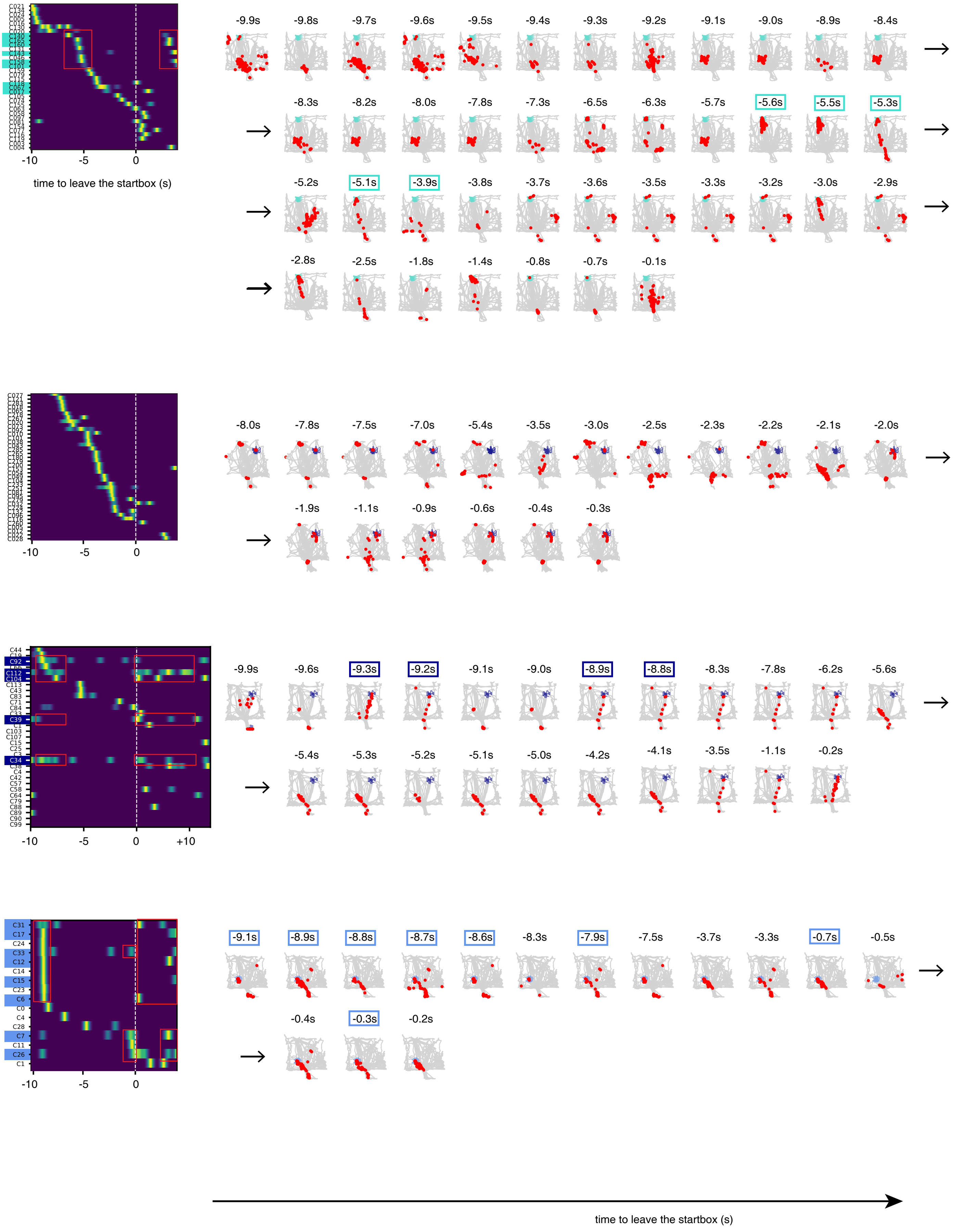
-10
-5
0
time to leave the startbox (s)
-9.9s -9.8s -9.7s -9.6s -9.5s -9.4s -9.3s -9.2s -9.1s -9.0s -8.9s -8.4s
-8.3s -8.2s -8.0s -7.8s -7.3s -6.5s -6.3s -5.7s -5.6s -5.5s -5.3s
-5.2s -5.1s -3.9s -3.8s -3.7s -3.6s -3.5s -3.3s -3.2s -3.0s -2.9s
-2.8s -2.5s -1.8s -1.4s -0.8s -0.7s -0.1s
-8.0s -7.8s -7.5s -7.0s -5.4s -3.5s -3.0s -2.5s -2.3s -2.2s -2.1s -2.0s
-1.9s -1.1s -0.9s -0.6s -0.4s -0.3s
+10
-9.9s -9.6s -9.3s -9.2s -9.1s -9.0s -8.9s -8.8s -8.3s -7.8s -6.2s -5.6s
-5.4s -5.3s -5.2s -5.1s -5.0s -4.2s -4.1s -3.5s -1.1s -0.2s
C31 C17 C24 C33 C12 C14 C15 C23 C6 C0 C4 C28 C7 C11 C26 C1
-9.1s -8.9s -8.8s -8.7s -8.6s -8.3s -7.9s -7.5s -3.7s -3.3s -0.7s -0.5s
-0.4s -0.3s -0.2s
time to leave the startbox (s)

Figure S17 Prospective coding. Examples of prospective coding from different sessions and animals as in Figure 3d. Highlighted are examples of identified sequences of cells coding the same prospective Sandwell. The time on top of the prospective coding content maps are the time before leaving to startbox (time=0)

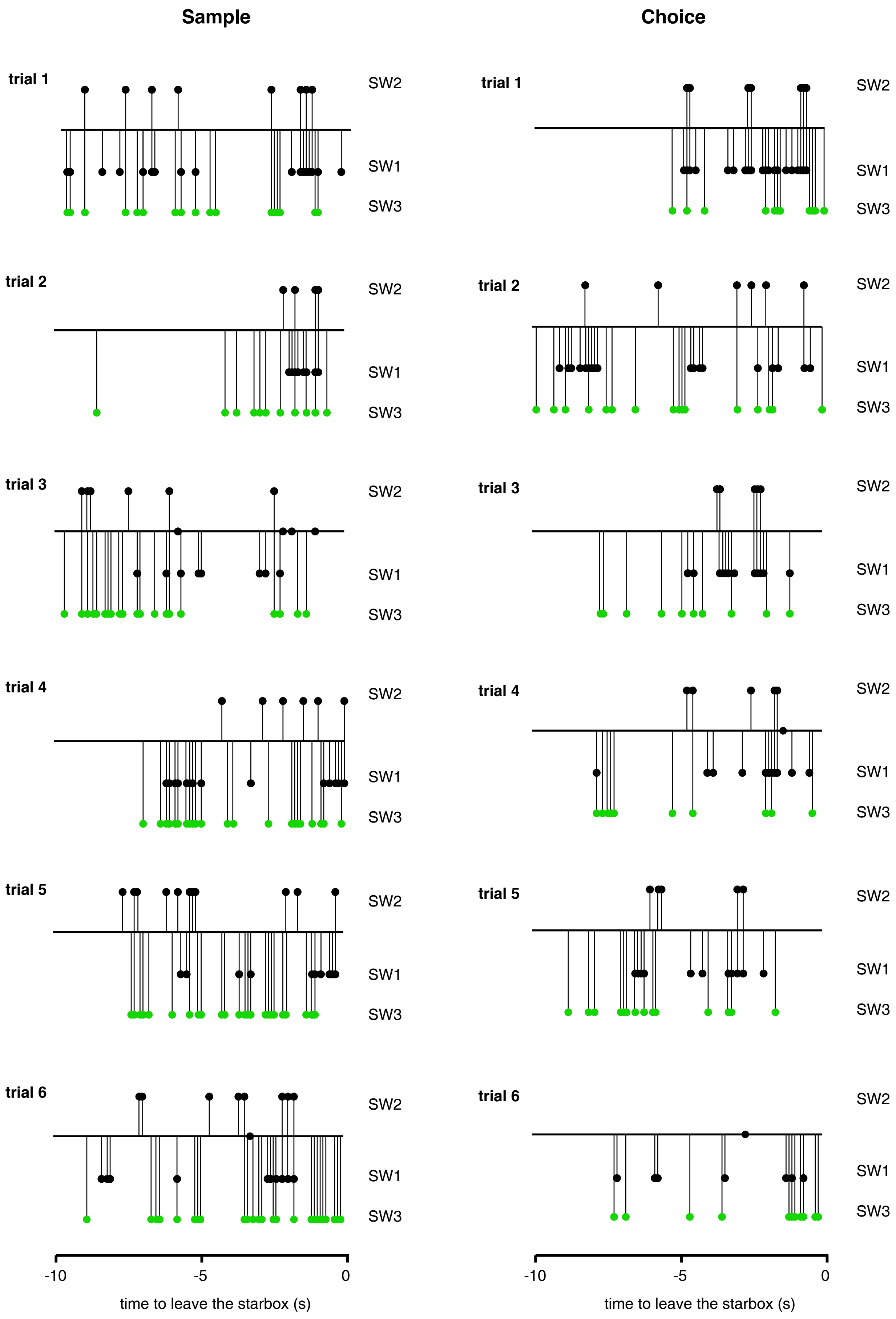
Sample
Choice
trial 1
trial 2
trial 3
trial 4
trial 5
trial 6
SW2
SW1
SW3
-10
-5
0
time to leave the starbox (s)

Figure S18 *Prospective coding*. Example of a whole session from the same animal showing the time and content of prospective coding events for the three sandwells for the 6 sample and 6 choice trials. Marks on the central line are the events marked as 'other' (see Methods).

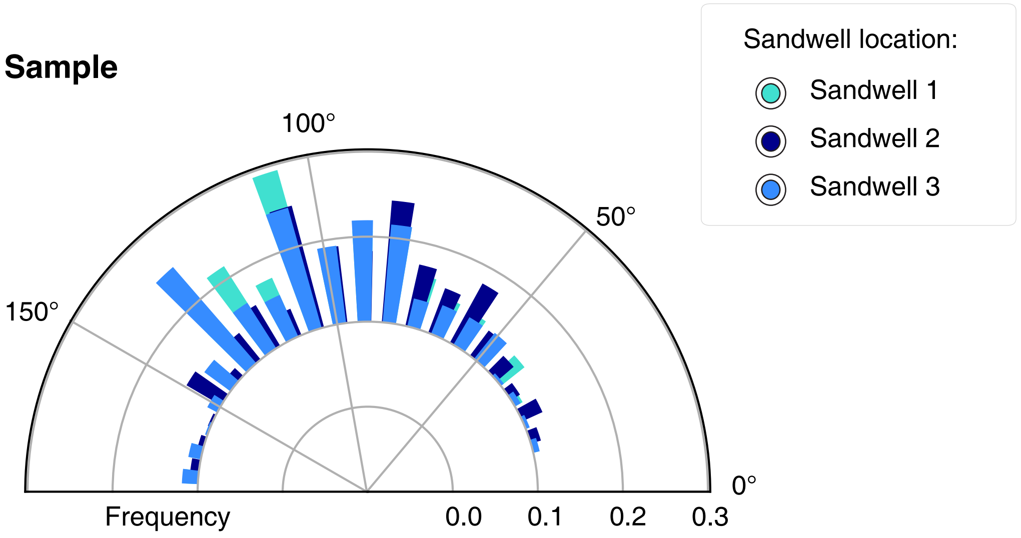

Figure S19 *Prospective coding*. Distribution of angular distances for cells during the 10s in Sample trials in the startbox as in Figure 3f.

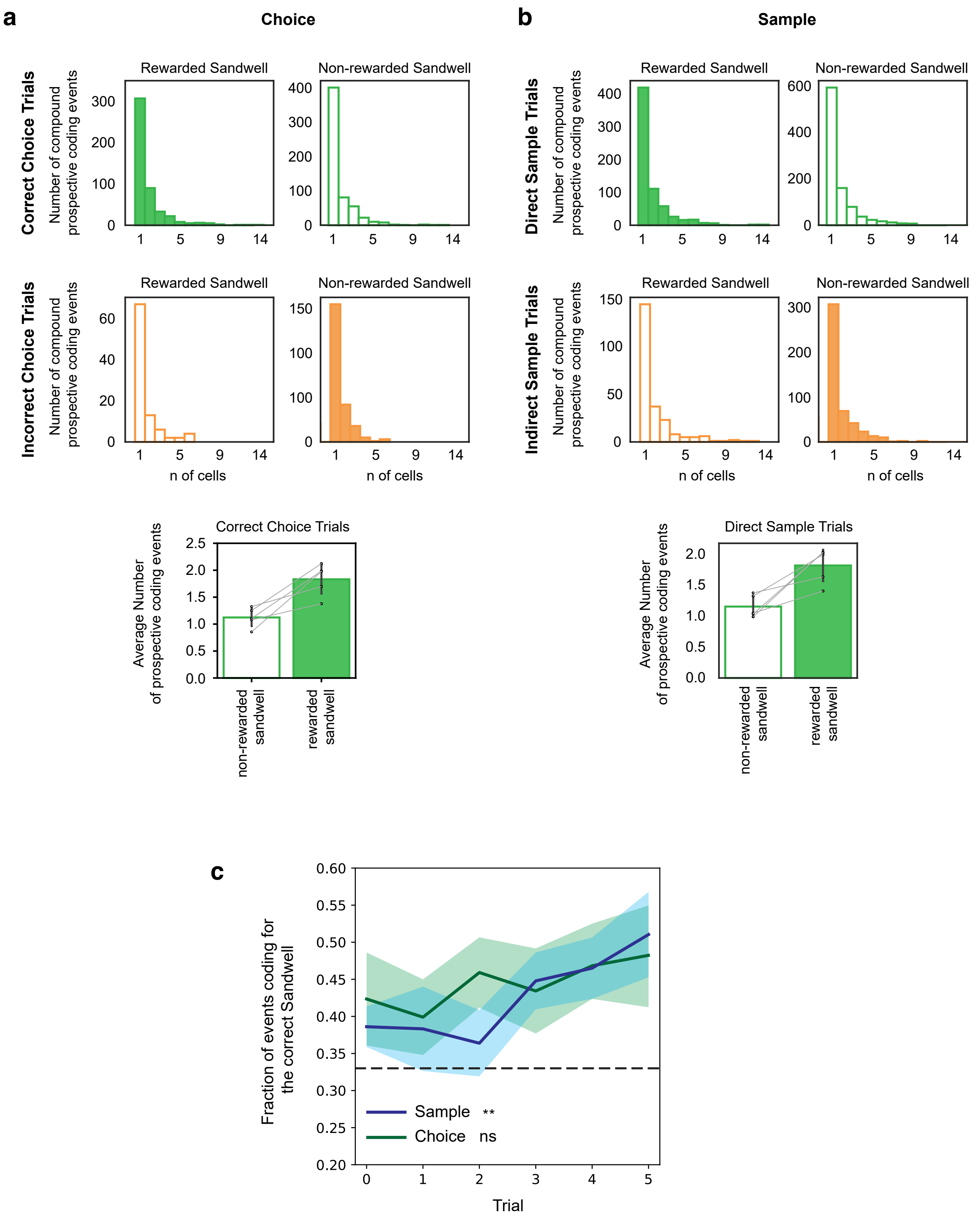

Figure S20 *Content of prospective coding*. Distribution of lengths of compound prospective coding events (number of cells) for choice (**a**) and sample (**b**) trials. (**c**) Fraction of events coding for the rewarded Sandwell divided by trial number for sample (blue) and choice (green) trials. Mean±sem. **P<0.001 one-way ANOVA test for trend.
